# Robust genome-wide ancestry inference for heterogeneous datasets: illustrated using the 1,000 genome project with 3D facial images

**DOI:** 10.1038/s41598-020-68259-w

**Published:** 2020-07-16

**Authors:** Jiarui Li, Tomás González Zarzar, Julie D. White, Karlijne Indencleef, Hanne Hoskens, Harry Matthews, Nele Nauwelaers, Arslan Zaidi, Ryan J. Eller, Noah Herrick, Torsten Günther, Emma M. Svensson, Mattias Jakobsson, Susan Walsh, Kristel Van Steen, Mark D. Shriver, Peter Claes

**Affiliations:** 1 Medical Imaging Research Center, MIRC, University Hospitals Leuven, Leuven, Belgium; 2 Department of Electrical Engineering, ESAT/PSI, KU Leuven, Leuven, Belgium; 3 Department of Anthropology, The Pennsylvania State University, University Park, PA USA; 4 Department of Neurosciences, Experimental Otorhinolaryngology, KU Leuven, Leuven, Belgium; 5 Department of Human Genetics, KU Leuven, Leuven, Belgium; 6 Murdoch Childrens Research Institute, Melbourne, VIC Australia; 7 Department of Biology, Indiana University-Purdue University Indianapolis, Indianapolis, USA; 8 Department of Organismal Biology, Uppsala University, Norbyvägen 18C, 75236 Uppsala, Sweden; 9 Medical Genomics Research Unit, GIGA-R, University of Liège, Liège, Belgium; 10 Walloon Excellence in Life Sciences and Biotechnology (WELBIO), Liège, Belgium

**Keywords:** Structural variation, Population genetics

## Abstract

Estimates of individual-level genomic ancestry are routinely used in human genetics, and related fields. The analysis of population structure and genomic ancestry can yield insights in terms of modern and ancient populations, allowing us to address questions regarding admixture, and the numbers and identities of the parental source populations. Unrecognized population structure is also an important confounder to correct for in genome-wide association studies. However, it remains challenging to work with heterogeneous datasets from multiple studies collected by different laboratories with diverse genotyping and imputation protocols. This work presents a new approach and an accompanying open-source toolbox that facilitates a robust integrative analysis for population structure and genomic ancestry estimates for heterogeneous datasets. We show robustness against individual outliers and different protocols for the projection of new samples into a reference ancestry space, and the ability to reveal and adjust for population structure in a simulated case–control admixed population. Given that visually evident and easily recognizable patterns of human facial characteristics co-vary with genomic ancestry, and based on the integration of three different sources of genome data, we generate average 3D faces to illustrate genomic ancestry variations within the 1,000 Genome project and for eight ancient-DNA profiles, respectively.

## Introduction

Scientists today have access to large heterogeneous datasets from many studies collected by different laboratories with diverse genotyping and imputation protocols. Therefore, the joint analysis of these datasets requires a robust and consistent inference of ancestry across all datasets involved, where one common strategy is to yield an ancestry space generated by a reference set of individuals^1^. Based on open-research initiatives such as the 1,000 Genome project (1KGP)^2^, HapMap project^3^, Human Genome Diversity project (HGDP)^4^, and the POPRES dataset^5^, the potential exists to create reference ancestry latent-spaces at different levels of interest, from worldwide inter-continental to fine-scale intra-continental ancestry. A reference ancestry space allows the researcher to collate multiple datasets facilitating analyses that are more advanced. For example, reference ancestry spaces can be used to infer the population structure of samples with family structure or cryptic relatedness^1^ and to investigate the genetic similarity between ancient DNA and modern human genomes^6^. They also have the potential to correct for population structure in a genome-wide association study (GWAS) on heterogeneous and admixed samples. Of final interest is the association of auxiliary data (e.g. specific phenotypes, such as 3D facial shape used in this work) present in internally collected datasets with ancestral variations captured by a reference space. This requires the projection of the collected datasets into a reference space, followed by an association of the projection scores with the auxiliary data presented.

Methodologically, the idea is to construct an ancestry latent-space from a reference dataset and to enable the projection of new cases from other datasets that follow the mainstream of the reference dataset. Starting from genome-wide single nucleotide polymorphisms (SNPs), principal component analysis (PCA) and analogous dimension reduction techniques on normalized genotype data are popular strategies used in this context^7,8^. However, in construction of an ancestry space, these approaches are known to be sensitive to outliers^7,9^. In addition and more importantly, in projecting new cases onto an ancestry space, PCA produces patterns of misalignment (for example, “shrinkage” patterns where projected cases tend to falsely gravitate towards the center of the ancestry space) due to missing data, missing heterozygotes, and genotyping along with imputation errors, which is misleading without careful interpretation^1^. Therefore, stringent quality control and data filters are typically in place to remove individual outliers and SNP data with high missing rates or not in Hardy–Weinberg equilibrium (HWE). However, in heterogeneous datasets, in contrast to homogeneous datasets, such data filters are harder to define, and potentially remove SNP data related to population structure. Furthermore, genotyping and imputation batch artefacts, not detected by quality control and different from one protocol to another, typically remain and still affect an integrative analysis of ancestry.

In this work, we propose a novel robust genome-wide ancestry inference (referred to as SUGIBS) based on the spectral (S) decomposition of an unnormalized genomic (UG) relationship matrix generalized by an Identity-by-State (IBS) similarity degree of individuals’ matrix. Robustness against outliers during ancestry space construction, is obtained by absence of specific sample statistics (e.g. allele frequencies). Furthermore, SUGIBS provides a robust projection of new samples, from different studies, onto a reference SUGIBS space. During projection, the IBS similarity degree of unseen individuals to individuals in the reference dataset acts as a correcting term for missing genotypes and errors, and most interestingly this correction is on an individual-by-individual basis. We test the robustness of SUGIBS and compare its performance to PCA and Multi-Dimensional Scaling (MDS) in revealing the underlying structure of an admixed population and adjusting for false positive findings in a simulated case–control admixed GWAS. Using the 1KGP as reference dataset, and an additional heterogeneous dataset containing 3D facial images, we apply SUGIBS to integrate the different datasets and combine this with a shape regression to construct average faces that illustrate the ancestral variations captured within the 1KGP. We further integrate eight ancient DNA profiles, which typically present higher genotyping error rates compared to modern DNA, and reconstruct their ancestry-derived average faces. Based on the results, our method facilitates a robust integrative analysis for ancestry estimation in heterogeneous datasets.

## Results

### Reference ancestry spaces with individual outliers

**Figure 1:**
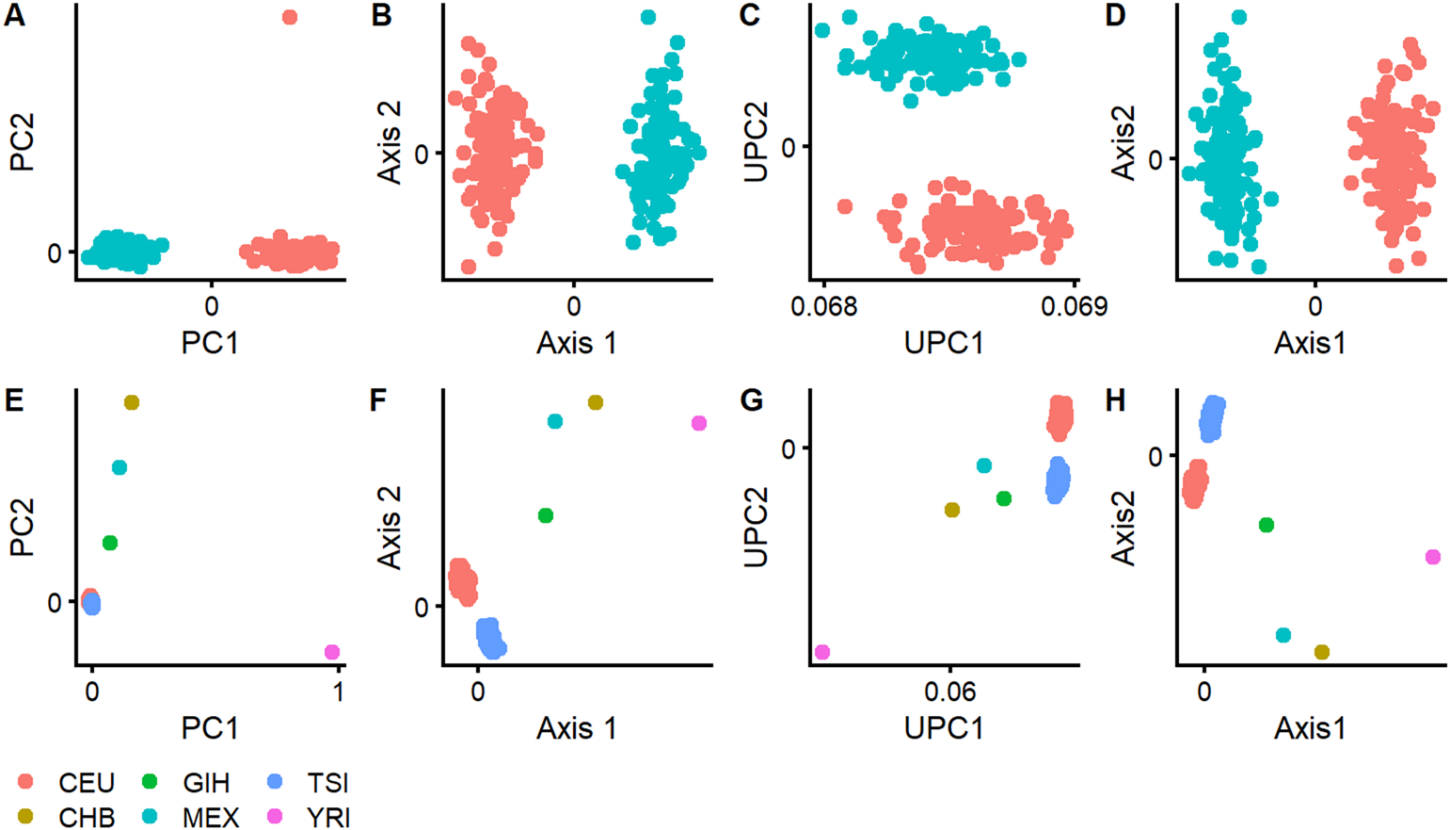
Robustness against individual outliers during the construction of an ancestry space. Top row, the first two ancestry components for (**A**) PCA, (**B**) MDS, (**C**) UPCA and (**D**) SUGIBS using the CEU and TSI populations from the HapMap 3 project. Bottom row, the first two ancestry components for (**E**) PCA, (**F**) MDS, (**G**) UPCA and (**H**) SUGIBS using the CEU and TSI populations from the HapMap 3 project, but with randomly selected single individuals from four different and additional populations (CHB, GIH, MEX and YRI) as “outliers”.

In the first experiment, we investigated the robustness of SUGIBS in comparison to traditional approaches, in particular PCA using normalized or unnormalized genotype data and MDS using IBS distances as they are implemented in PLINK 1.9^10^, against individual outliers in a reference dataset. For this purpose, we first selected all unrelated individuals from the CEU and TSI populations in the HapMap 3 project^3^ and used SUGIBS, PCA, unnormalized PCA (UPCA) and MDS to illustrate the first and second latent dimensions as ancestry components (Fig. 1, top row). In contrast to the traditionally used normalized genotypes in PCA, UPCA used unnormalized genotypes that were not centralized around the mean and were not standardized to a variance equal to one. As expected, PCA, MDS and SUGIBS are able to differentiate between both populations along the first ancestry component. UPCA differentiates between both populations along the second ancestry component, with the first aggregating an average SNP pattern, which is common when running PCA on non-centered data. Surprisingly, with PCA a single outlier (NA11917) that was not expected during the selection of both populations already affected the second ancestry component. Subsequently, we randomly selected one individual from four different and additional populations (CHB, GIH, MEX and YRI) as “true outliers” in the dataset. Figure 1, bottom row, illustrates the first two ancestry components of the four methods constructed on the dataset with outliers, where all four approaches clearly separate the outliers. Using PCA, in contrast to MDS, UPCA and SUGIBS the clear distinction between CEU and TSI is lost within the first two ancestry components, as they mainly capture variations due to the outliers. The main reason for robustness in UPCA, MDS and SUGIBS is that these three methods use unnormalized genotype data and therefore do not rely on specific sample statistics (e.g. allele frequencies), that otherwise increase the influence of outlier variation. The single outlier (NA11917) in Fig. 1(A) using PCA disappears when true outliers were added to the data in Fig. 1(E). This indicates that the population label CEU of individual NA11917 is most likely correct in the context of variation present from multiple populations. Therefore, NA11917 is likely a false outlier, potentially due to small errors in the data of this individual, but this is harder to verify.

### Robust projection of unseen individuals onto reference ancestry spaces

**Figure 2:**
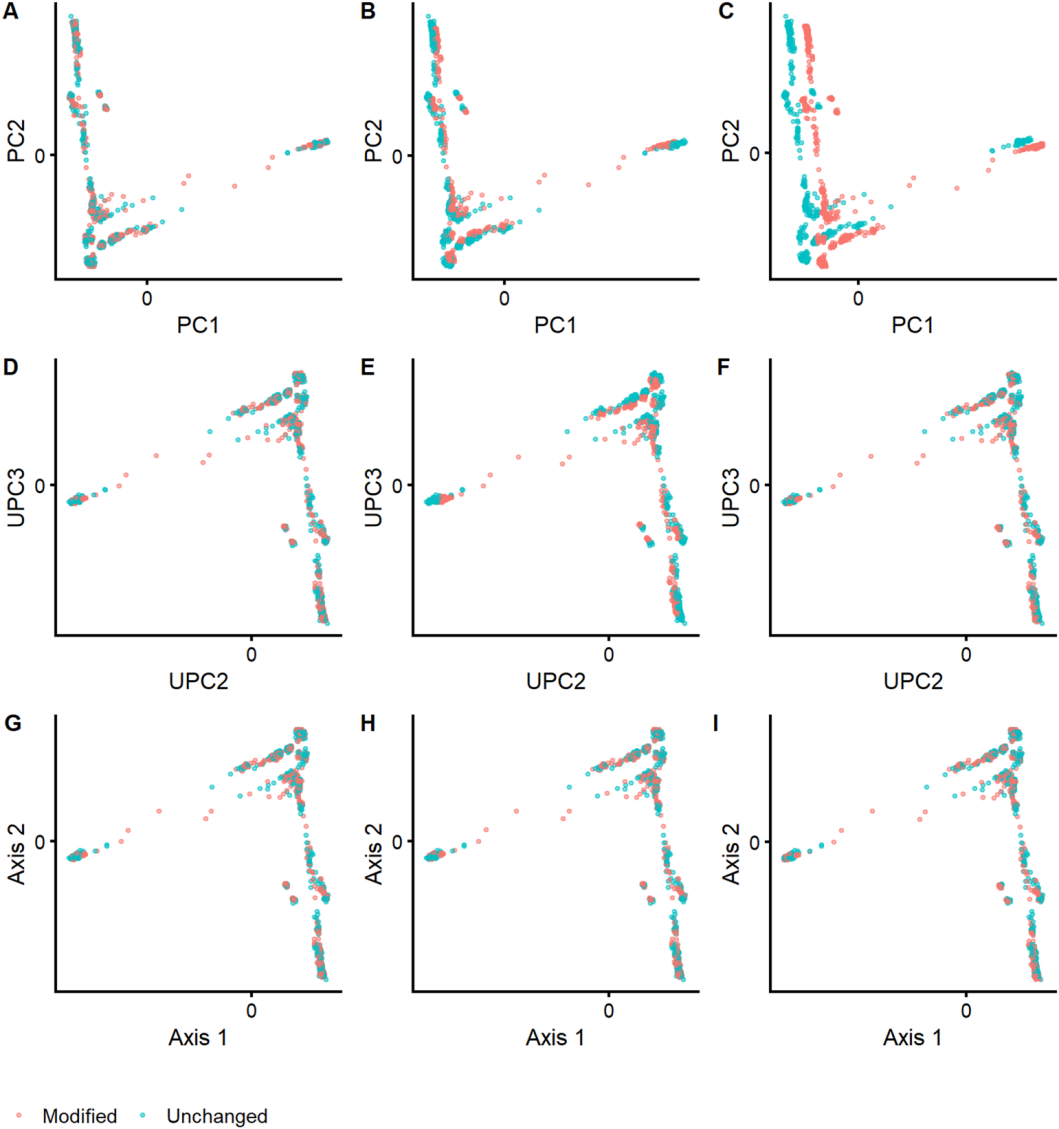
Robustness against batch artefacts during the projection of two distinct HGDP data partitioned samples, one to modify (pink) and one to remain unchanged (blue), onto a 1KGP based reference ancestry space. First Column, (**A**), (**D**), and (**G**), no real modification of the genotypes was done whereby both data partitions, pink and blue, overlap perfectly for PCA, UPCA and SUGIBS, respectively. Second column, (**B**), (**E**), and (**H**), a first actual modification was done by introducing missing genotypes in the data of the partition to modify (pink) with results for PCA, UPCA and SUGIBS, respectively. Third column, (**C**), (**F**), and (**I**), a second actual modification was done by modifying genotypes in the data of the partition to modify (pink) with results for PCA, UPCA and SUGIBS, respectively.

**Figure 3:**
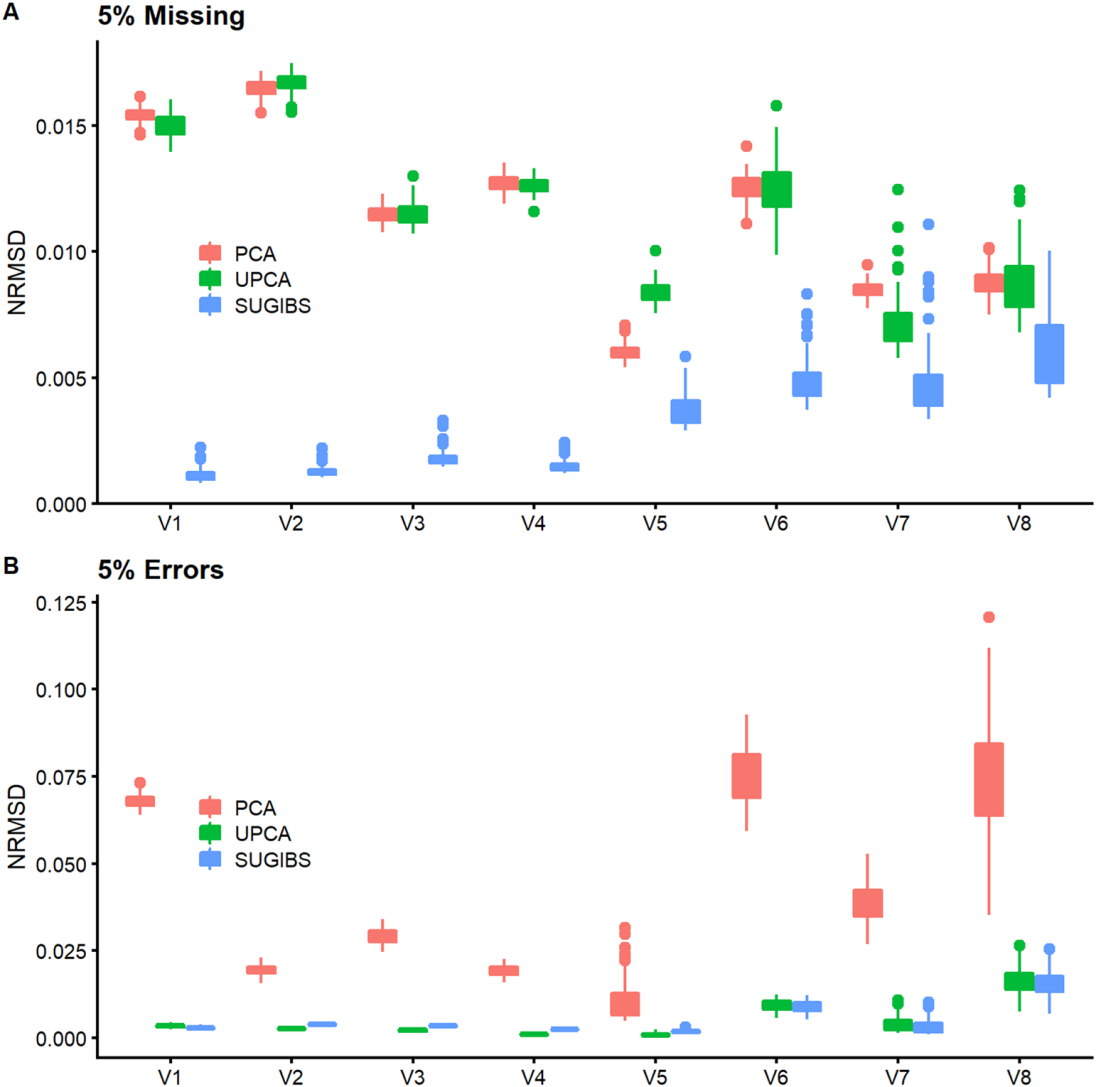
Normalized root-mean-square deviation (NRMSD) of the top eight axes of PCA, UPCA and SUGIBS. NRMSD measures the root-mean-square differences (RMSD), for the modified HGDP dataset only between the scores on ancestry axes generated using the original genotypes (error free) and the modified genotypes (with simulated errors, **A**) missing genotypes and (**B**) erroneous genotypes). The RMSD values were normalized by the range of the ancestry axes generated using the original genotypes, so that NRMSD of the three methods (PCA, UPCA and SUGIBS) are comparable.

In a second experiment, we projected (“Methods”, Eq. 4) new samples on an ancestry space, based on the 1KGP as reference dataset, to investigate the robustness of SUGIBS in comparison to PCA and UPCA against typical artifacts of different laboratory protocols. Note that, since the first component of UPCA just aggregated the average SNP pattern as seen in experiment 1, we started UPCA from the second component onwards. MDS does not allow for a straightforward projection of new samples on a reference space and was therefore excluded. As samples to project, we randomly assigned all 1,043 individuals of 51 populations from the HGDP dataset^4^ into two equally-sized samples, one unchanged and one modified, respectively. To investigate the influence of different rates of missing data, we randomly masked 5% of the SNP genotypes as missing in the modified dataset (See “Methods”). For the influence of different rates of errors, we partially changed SNP genotypes with minor allele frequency (MAF) less than 5% in the modified dataset (See “Methods”). Note that this was done knowing that more imputation errors are observed in SNPs with a MAF of 5% and less^11^. We projected both HGDP datasets onto the PCA, UPCA and SUGIBS reference spaces as defined by the 1KGP. In PCA, the simulated artefacts generated “shrinkage” and “shifting” patterns of misalignment in the first two projected ancestry components (Fig. 2, top row), for missing and erroneous genotypes, respectively. UPCA was only influenced by missing genotypes (Fig. 2, middle row). In contrast, SUGIBS was not influenced by missing or erroneous genotypes (Fig. 2, bottom row). Figure 3 summarizes the normalized root-mean-square deviations (NRMSD) of the first eight axes of SUGIBS, UPCA and PCA of the modified HGDP dataset over 100 simulations. SUGIBS is significantly more robust than PCA in the presence of missing and genotyping/imputation errors in new data for which ancestry needs to be inferred, by projecting it into a reference space.

### Revealing and adjusting for population structure in an admixed population

**Figure 4:**
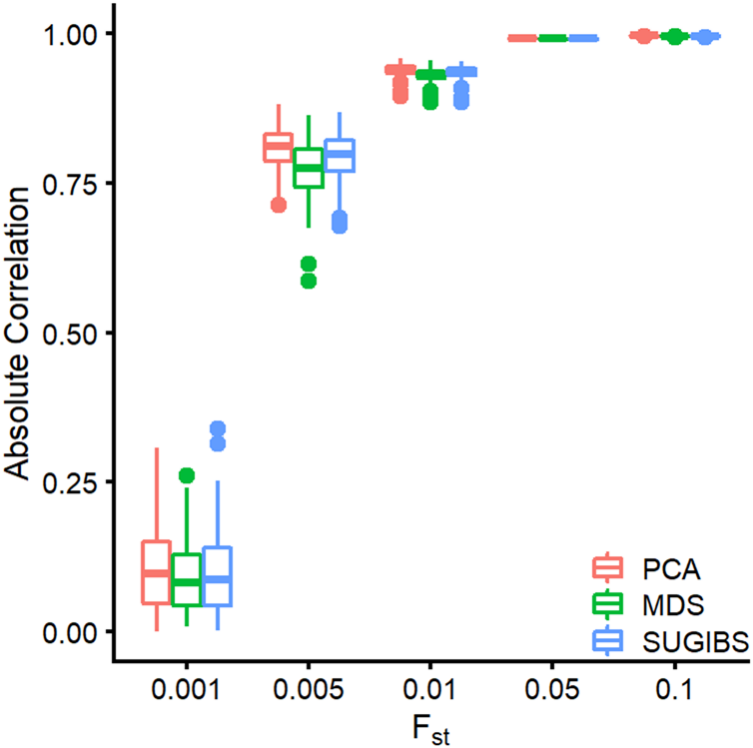
Capturing simulated admixture in function of ${\mathrm{F}}_{\mathrm{s}\mathrm{t}}$. X-axis represents the different levels of Fst investigated. The Y-axis represents the absolute correlation of the first component in PCA, MDS and Spectral-IBS with the simulated ancestry proportion. The higher the correlation the better a method is able to capture the underlying admixture.

In a third experiment, we investigated the ability of SUGIBS compared to PCA and MDS in representing admixture, since both are commonly used in practice. UPCA on the other hand is not commonly used. However, it shares a strong overlap with SUGIBS in starting from unstandardized genotypes, and therefore the results of UPCA are not reported here since they corroborate perfectly with those of SUGIBS. Following the work of Galinsky et al.^12^, we simulated data at 3,200 random independent SNPs for 200 individuals from a recent admixture of two populations, 50% from each population on average with divergences ${F}_{st}=\{0.001, 0.005, 0.01, 0.05, 0.1\}$, from an intra-European difference to an intercontinental difference^13^. Because the admixture contains only one dimension of population structure, only the first component of variation is of interest. Figure 4 presents the absolute correlations between the first component of PCA, MDS and SUGIBS and the simulated ancestry proportions over 100 runs. When the ${F}_{st}$ divergence between two populations is lower than 0.05, the correlation between the SUGIBS component and the ancestry proportion is similar to that of MDS, but a little lower than PCA. We noticed that when ${F}_{st}\le 0.01$, all three methods have a reduced performance to reveal the underlying admixture and when ${F}_{st}>0.01$, all three methods perform perfectly. According to the work of Patternson et al.^8^, the theoretical finest divergence between two isolated populations, which can be detected by this amount of SNPs and samples would be ${F}_{st}=\frac{1}{\sqrt{3200\times 200/2}}\approx 0.002$. In line with this theoretical boundary, our results show that for ${F}_{st}=0.001$, all methods hardly detected any structure. At ${F}_{st}=0.005$, all three methods start to detect population structure ($\left|r\right|>0.7$).

Following the work of Price et al.^14^, we also simulated a case–control GWAS to investigate if the population structure inferred by SUGIBS can be used for correcting population stratification as a confounder. Only low divergences between the two populations ${F}_{st}=\{0.001, 0.005, 0.01\}$, were tested, because for larger divergences all three methods would perform the same as deducted from the previous experiment. Tests were conducted with a logistic regression under four different correction scenarios: (1) no population for stratification correction (Naïve), (2) PCA, (3) MDS and (4) SUGIBS, using a likelihood ratio test for the significance of each genetic marker. The experiment was conducted 100 times, using 1 million SNPs on 1,000 individuals, with average proportions of SNPs detected as significant shown in Table 1. These results indicate that in a single dimensional population structure, correcting using MDS, SUGIBS and PCA perform similarly, both in terms of Type I error and power. All three methods failed to correct the population stratification when ${F}_{st}=0.001$, which is consistent with the failure of the three methods in revealing the admixture structure in the previous experiment. Finally, these results are in line with the results in the work of Price et al.^14^.

**Table 1:** Proportion of associations reported as statistically significant ($\mathrm{P}<0.0001$) by logistic regression using a likelihood ratio test.

	Naive	PCA	MDS	SUGIBS
$${{\varvec{F}}}_{{\varvec{s}}{\varvec{t}}}=0.001$$				
Random	0.0002	0.0001	0.0001	0.0001
Differentiated	0.9960	0.4483	0.6370	0.5200
Causal	0.5295	0.4779	0.4865	0.4807
$${{\varvec{F}}}_{{\varvec{s}}{\varvec{t}}}=0.005$$				
Random	0.0009	0.0001	0.0001	0.0001
Differentiated	0.9980	0.0002	0.0003	0.0002
Causal	0.5226	0.4249	0.4255	0.4253
$${{\varvec{F}}}_{{\varvec{s}}{\varvec{t}}}=0.01$$				
Random	0.0030	0.0001	0.0001	0.0001
Differentiated	0.9971	0.0001	0.0001	0.0001
Causal	0.5166	0.4227	0.4230	0.4229

Random SNPs with no association to the disease were generated by simulating random drift with ${\mathrm{F}}_{\mathrm{s}\mathrm{t}}$ divergence. Differentiated SNPs with no association to the disease were generated by assuming population allele frequencies of 0.8 of ancestry 1 and 0.2 of ancestry 2. Causal SNPs were generated by combining a multiplicative disease risk model while simulating the random drift with the same ${\mathrm{F}}_{\mathrm{s}\mathrm{t}}$ as the random SNPs. See methods for more details on the parameters.

### Practical illustration of genome data integration using 3D facial images

**Figure 5:**
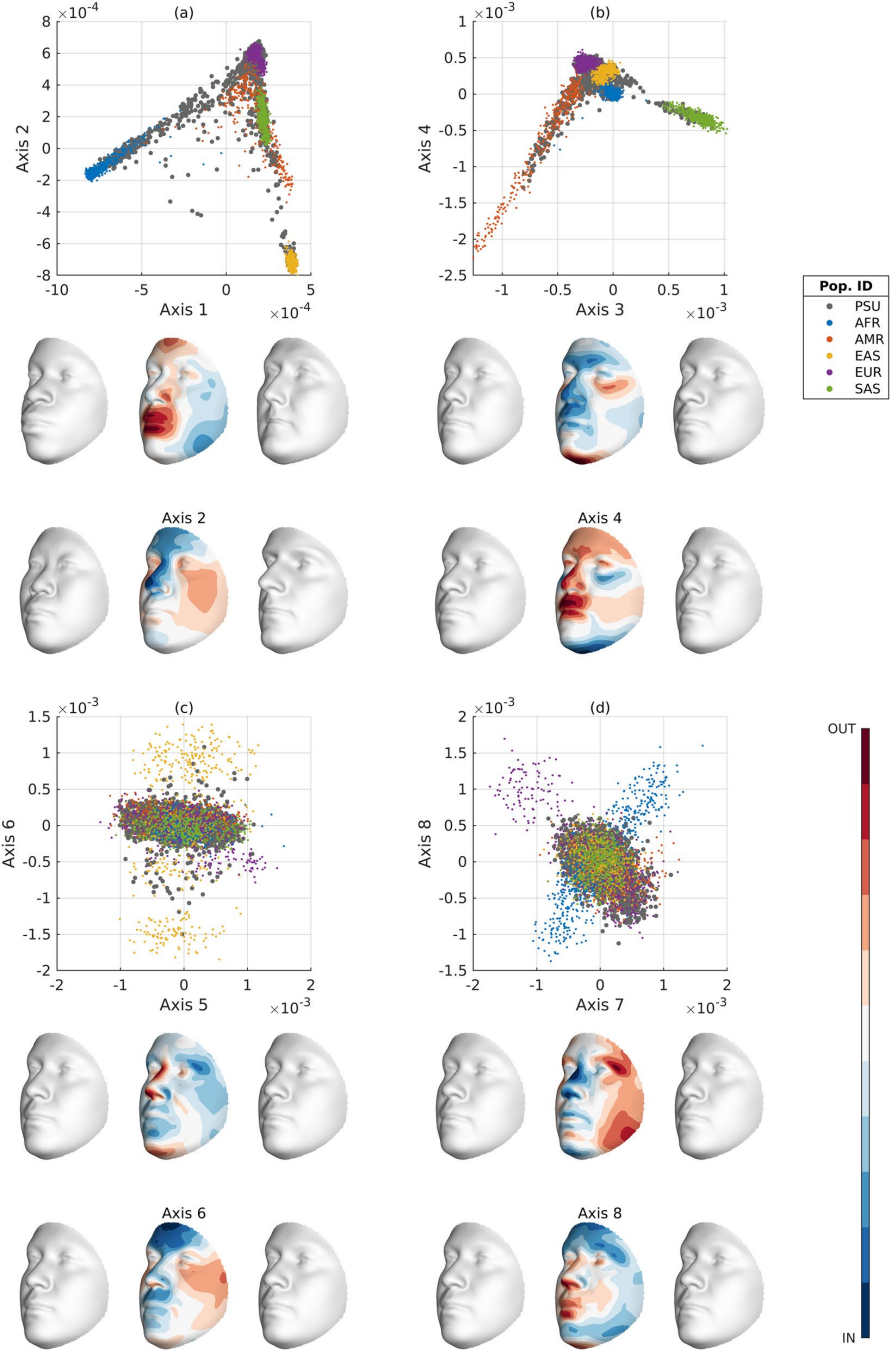
Top eight SUGIBS axes of 1KGP and projections of the PSU cohort. (**A**) The first (horizontal) and second (vertical) axes. (**B**) The third (horizontal) and fourth (vertical) axes. (**C**) The fifth (horizontal) and sixth (vertical) axes. (**D**) The seventh (horizontal) and eight (vertical) axes. Grouped populations of the 1KGP are coloured dots (AFR, African, AMR, Admixed American, EAS, East Asian, EUR, European, SAS, South Asian). The projected PSU cohort are represented by grey dots. The faces illustrate opposing variations along each of the ancestry components and are not associated to any of the 1kG populations in particular. For each axis, we visualize a face − (left) and + (right) 3 times the standard deviation (sd) from the 1KGP mean on that axis. In the middle, the blue-red colormap highlights inward (blue) and outward (red) facial difference going from the + 3 sd to the − 3 sd facial gestalts. The values for sex, height, weight and age were 1.5 (genderless), 165 cm, 75 kg and 30 years, respectively.

Putting SUGIBS to practice in integrating multiple datasets, we projected 2,882 unrelated individuals from a large admixed and heterogeneous dataset containing individuals from varying ancestries (the PSU cohort, see Methods) and eight famous ancient DNA samples onto the first 25 SUGIBS axes established from the 26 populations in the 1KGP. Using the first eight SUGIBS scores of the PSU cohort onto the ancestry components of the 1KGP, we fitted a partial least squares regression (PLSR) to model facial variations in function of each of the first eight ancestry components. Shown in Figs. 5 and S1–5(a), the first two ancestry components separate the African (AFR) and East Asian (EAS) populations from the remaining populations, as indicated by the population labels given in the 1KGP. The next two ancestry components in Figs. 5 and S1–5(b) separate the South Asian (SAS) population and visualizes the admixture in the Admixed American (AMR) population, respectively. In Figs. 5 and S3(c), the sixth ancestry component captures different subpopulations in the EAS population. In Figs. 5 and S1(d), the seventh ancestry component is driven by African subpopulations. The separated European subpopulation on the eighth ancestry component is the population from Finland (FIN), Fig. S4(d). The projected PSU cohort is indicated by gray dots in Figs. 5 and S1–5 and overall it is observed that they overlay well with a wide range of ancestry variations in the 1KGP confirming the heterogeneous and admixed nature of the PSU dataset. However, some populations in the 1KGP are less covered by the PSU cohort, such as the population of Finland in Europe and some African subpopulations on ancestry components seven and eight (Fig. 5d).

**Figure 6:**
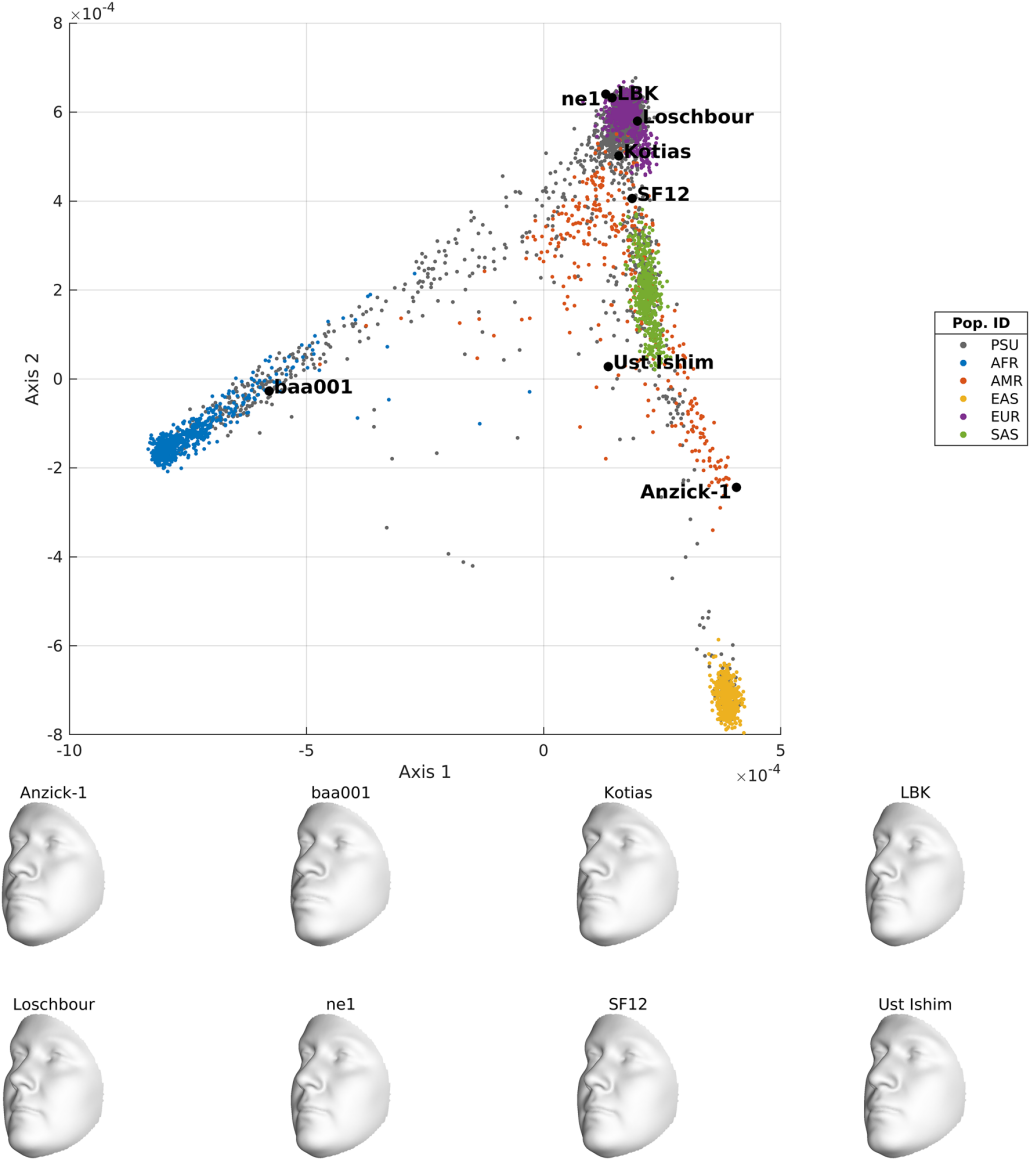
Ancestry-derived facial averages based on eight ancient DNA profiles. For these facial averages, the sex (1 for male, 2 for female) was known from the DNA profile and taken into account in the partial least squares regression model. The values for height, weight and age were 165 cm, 75 kg and 30 years, respectively.

Based on the visually strong and recognizable human facial phenotype, we generated comprehensive illustrations of the population structure embedded in the 1KGP. In Fig. 5, strong facial differences are observed for ancestry components 1–4, whilst perceptually smaller differences occur in components 5–8. This is most likely due to a lower overlap of the PSU cohort with these ancestry components. i.e. the PSU cohort captures the variation across the larger continental population groups, but not the variation at the finer intra-continental population level. Finally, in integrating additional data sources from ancient DNA into the same ancestry space we derived an ancestry-driven average facial representation for eight high-coverage ancient DNA profiles (Fig. 6). It is observed that their projections within the 1 KGP ancestry space is consistent with the intercontinental geographical locations where these samples were discovered and what is currently known about these samples (Supplementary Table S1).

## Discussion

Accurate inference of population structure and individual global ancestry is of critical importance in human genetics, epidemiology, and related fields^15,16^. The analysis of population structure in itself can yield significant insights in terms of population dynamics, both in modern and ancient populations^17–19^. Through inspection of ancestry components as well as distances in genetic latent spaces created by, for example, principal component analysis (PCA), it is possible to infer patterns of gene flow and population movements through time. Furthermore, The inclusion of various populations in genome-wide association studies (GWAS) could increase statistical power and make better contributions to our understanding of the genetics of complex traits for the human population as a whole^20^. However, the widely used approach of PCA and analogous techniques are sensitive to outliers, when constructing ancestry spaces, and produce patterns of misalignment due to artifacts of different laboratory protocols when new samples are projected onto a reference ancestry space^1,7,9^. We propose a robust alternative for genome-wide ancestry inferencing, referred to as SUGIBS. Our results confirm the erroneous influences in PCA based ancestry estimations that are misleading without careful interpretation. In constructing an ancestry space, SUGIBS shares the same robustness against individual outliers as MDS or related spectral graph approaches^21^. Furthermore, and more importantly, during dataset projections SUGIBS is robust against typical artefacts from different laboratory protocols. In addition, SUGIBS achieved almost the same performance, under error-free conditions, as PCA in revealing the underlying structure of an admixed population and avoiding false positive findings in a simulated case–control GWAS with an admixed population.

Irrespective of the method of choice, two general notes apply here. First, as with MDS and PCA, there is a range of possible population histories that could generate the same pattern in SUGIBS. For example, a gradient including multiple individuals could be interpreted as the result of gene flow, admixture or isolation-by-distance. In such situations, it is important to see methods like PCA and SUGIBS as exploratory analysis only to generate hypotheses which can and should be formally tested using additional approaches (e.g. f statistics for gene flow and admixture^22^). Second, although detecting and adjusting for population stratification in closely related populations and hybrids of these are more difficult than for populations that show more differentiation (higher F_st_), there are important questions regarding how much such stratification can affect studies performed in what are considered "homogeneous" populations with respect to admixture; in other words, cryptic stratification^23^. It will also be important to understand how well cryptic stratification can be controlled by the various methods as a function of minor allele frequency, i.e., rare SNP vs. common SNP^23^.

Like MDS and SUGIBS, PCA is also a “spectral” method, in which the edge similarity between individuals is simply the covariance of normalized genotypes, commonly referred to as the genomic relationship matrix^24^. However, this covariance similarity used in PCA depends on the allele frequencies as a non-robust sample statistic to normalize the genotypes, which causes sensitivity to individual outliers. Note that in our experiments on PCA without using allele frequencies (UPCA) robustness against individual outliers was observed. Among the “spectral” methods, some other robust alternatives were introduced to infer population structure, including a modified genomic relationship^21,25^. MDS or related spectral graph approaches^21^ using IBS and Allele Sharing Distance (ASD) similarities between individuals (available in PLINK^10^) are also a robust alternative against individual outliers, as illustrated in our results. IBS and ASD are unnormalized distances, and thus less influenced by outliers. However, MDS and the modified genomic relationship^21,25^, both lack the ability to project new samples on an already established reference ancestry space. Alternatively, it might be possible to use one of the many robust PCA approaches that have been investigated for general data^26–28^ as well as genetic data^29^. However, in most study data processing protocols, robust approaches are usually used for outlier detection rather than inferring population structure, which is done by classical PCA after excluding outliers^29^. This is for example, a standardly used option in the popular EIGENSOFT software^7^. Note that, when establishing an ancestry space from a reference dataset, it remains good practice to identify and remove individual outliers, if they are of no further interest.

The main contribution of SUGIBS is robustness against batch artifacts of different laboratory and data processing protocols when projecting new samples onto a reference ancestry space. In the case of missing genotypes, smaller absolute PC scores, and smaller UPC scores are wrongfully generated during the projection of samples. These smaller and decreased scores lead to the “shrinking” and “shifting” patterns as observed in the results. (Note that this is not to be confused with PCA shrinkage due to high dimensional and large-scale data, which is dealt with using shrinkage eigenvalue estimations as recently implemented in EIGENSOFT). However, to correct for this, the projected SUGIBS score matrix is weighted by the reference degree matrix, which captures the similarity between the data to be projected and the reference data (see “Methods”). This weighting of projected SUGIBS scores equally corrects for the effects of genotyping and imputation errors, as demonstrated in the results. To the best of our knowledge, we are currently not aware of another related approach that offers the same robustness. Based on the results, we argue that SUGIBS is a solid alternative to PCA and MDS and requires less stringent data filters to operate. Our implementation of SUGIBS uses the randomized singular value decomposition algorithm^30^, that is also used in FastPCA^12^. This makes the algorithm computationally tractable for datasets with tens of thousands of individuals and millions of SNPs. E.g. the calculation of the loadings for eight components based in 6,000 individuals using 300,000 SNPs takes two minutes compared to one hour using a standard singular value decomposition on a Mac Pro (Late 2013), Intel Xeon CPU E5-2697 v2 @ 2.70 GHz (12 core), 64.00 GB 1,600 MHz DDR3. SUGIBS is available as part of an open-source in-house matlab library, referred to as SNPLIB, in which we used PLINK binary file formats as input, and provide FastPCA, logistic GWAS and all other methods, including the simulations mentioned throughout this work. Furthermore, SUGIBS can easily be incorporated into existing and interesting extensions to derive common ancestry estimations in datasets with non-overlapping genetic variants^1^, or genotyping-by-sequencing data^31^, or population structure inference in presence of relatedness^32^, or in iterative schemes to obtain global to fine-scale ancestry estimations^33^.

There are a few points of discussion and future investigations. First, a genetic similarity measure between pairs of individuals aims to identify how they are related and different measures exist for ancestry estimations (e.g. IBS, ASD, Identity-by-descent, normalized covariance)^24^. Commonly used similarity measures are normalized, just like the traditional approach of PCA on normalized genotype data, to take the genetic composition of individuals along with the rest of the sample into account. A normalization does have the advantage that individuals within the same population are more similar to each other than to individuals in other populations^24^. In other words, the distinction between populations increases, which improves population identification by clustering algorithms. However, when the normalization is performed incorrectly clustering efforts might be inaccurate. Furthermore, as seen in our results, such a normalization increases the influence of individual outliers. Finally, in contrast to homogeneous datasets, normalization of genotype data in heterogeneous datasets is challenging depending on whether the dataset is unlabeled or not, imbalanced or not, and with high admixture or not. Starting from unlabeled data, unsupervised clustering approaches such as ADMIXTURE^34^ and STRUCTURE^35^, iteratively identify the populations individuals belong to and update the normalization accordingly. However, this involves additional parameters to set and tune, the most important one being the amount of clusters expected in the data. Without prior knowledge on how to set these parameters, this can turn into a challenging task. With highly admixture data, any clustering of global ancestry into populations is even questionable. In these situations, only local ancestry estimations, using chromosome painting approaches^36^ for example, are meaningful. Alternatively, in the future, we want to investigate the use of a reference ancestry space as constructed in this work, without assigning individuals to specific populations, in estimating normalized genotype data on an individual-by-individual basis. I.e., an ancestry space from unnormalized genotype data is a good first step unbiased by any sample statistics, to further deduct statistics related to individual genotype profiles. For example, Kwong et al.^37^ propose the Robust Unified Test for Hardy–Weinberg Equilibrium in the context of an admixed population, which also makes use of individual-level adjustments for ancestry. Second, future investigations of the methodology also include the influence of Linkage Disequilibrium (LD) pruning and data filtering for SNP selection. Population admixture is one of the main sources for LD between SNPs, therefore we prefer to avoid excessive LD pruning before applying SUGIBS. As stated in Lawson and Falush^24^ any data pruning or filtering is bound to loose information related to population structure. For example, less common variants are typically lost in data filtering, but these might contain valuable information about population structure^24^. Since SUGIBS is robust and computationally tractable, any data filtering can be minimized. Third, another future investigation involves the determination of the number of relevant or significant components in SUGIBS, for which we provide a preliminary suggestion that compares the spectrum of the data observed with that of a simulated homogenous dataset assuming linkage equilibrium (LE) and Hardy–Weinberg Equilibrium (Supplementary Note S1).

We recommend the following procedure to extract a common set of SNPs between a reference dataset and another dataset being projected, for constructing SUGIBS ancestry spaces. First, exclude all the indel, monomorphic, and multi-allelic SNPs in both the reference dataset and the dataset to project. Subsequently, extract the list of SNPs common in both datasets. Based on this list, we further recommend a minor allele frequency (MAF) filtering with a MAF threshold of 1% on the reference dataset using PLINK^10^ as a quality control step. We do not recommend Hardy–Weinberg disequilibrium (HWD) filtering since it is probably the result of population admixture and thus useful for our purposes^38^. Although population admixture is one of the main sources for LD between SNPs, we still recommend LD pruning since it is not unusual to have non-uniformly genotyped genomes. Similar to PCA, SUGIBS does not explicitly model LD between SNPs so that misleading results might be generated without LD pruning.

In application of SUGIBS we used the human face, which is a powerful phenotype to visualize and illustrate underlying genetic ancestry variations. Indeed, faces are easy to recognize, interpret, and validate the outcomes based on everyone’s expert knowledge in facial perception. The faces illustrating the variation in ancestry components of the 1KGP in this work overlay well with the 1KGP super population labels that represent intercontinental differences. Facial variations at the level of intracontinental populations were perceptually harder to observe, mainly due to a lack of overlap of the PSU cohort with many of the 26 1KGP subpopulations. We further used SUGIBS to integrate eight ancient DNA profiles into the 1KGP ancestry space. Despite many advances in ancient DNA methodologies, DNA extracted from archaeological remains is usually associated with higher error rates than modern DNA. Over time, different biochemical processes cause a degradation of the DNA molecules leading to short fragments^39^ that increase the chances of mapping errors. Furthermore, post-mortem deamination of cytosine into uracil is generating false cytosine to thymine changes after PCR and sequencing which increases the per-sample error rate^40^. Furthermore, handling of human remains by other human individuals comes with the inherent risk of modern human contamination which can be seen as another source of errors introduced into the DNA sequence of the ancient individual. Therefore, even though any other method, such as PCA, can be used in a shape regression framework to generate average ancestry 3D faces, SUGIBS provides a “piece of mind” against the inevitable presence of genotyping errors and missing data as illustrated throughout the results. Finally, it is important to note that an ancestry-derived face are not individually specific faces, but average faces that simply visualize the ancestry background of a DNA profile. Related work on facial prediction from DNA^41,42^, also show that sex and ancestry are primary factors driving the estimation of facial shape from DNA. Further information on the potential value of ancestry-derived faces is provided in Supplementary note S2.

In conclusion, SUGIBS is a novel approach to construct an ancestry space from a reference dataset and to project new samples from heterogeneous datasets for a consistent and robust inference of individual ancestry. The main contributions involve robustness against outliers during the construction of an ancestry space, and robustness against batch artefacts during the projection of new samples into an ancestry space. Therefore, SUGIBS is a solid alternative to PCA and MDS and facilitates a robust integrative analysis for population structure and ancestry estimations for heterogeneous datasets. Based on the visually strong and recognizable human facial phenotype, comprehensive illustrations of genomic ancestry related variations within the 1KGP and for eight ancient-DNA profiles were provided.

## Materials and methods

### SUGIBS reference-space

Given a dataset with $N$ individuals and $M$ SNPs, we first create an unnormalized genotype (UG) matrix ${{\varvec{X}}}_{M\times N}$ with additive genotype coding (*aa* = − 1, *Aa* = 0, *AA* = 1 and missing = 0). The UG relationship matrix is then defined as ${\varvec{G}}=\frac{1}{M}{{\varvec{X}}}^{T}{\varvec{X}}$. From ${{\varvec{W}}}_{N\times N}$, the IBS similarity matrix of the same dataset used to create ${\varvec{G}}$, the similarity degree of an individual can be defined as ${d}_{ii}={\sum }_{j=1}^{N}{w}_{ij}$. We followed the algorithm implemented in PLINK to calculate the IBS similarity so that:

**Table:** 

IBS	AA	Aa	aa
AA	2	1	0
Aa	1	2	1
aa	0	1	2
N/A	0	0	0

However, in contrast to the calculations in PLINK, we do not normalize the IBS similarity matrix with missingness scores. This results in a similarity degree matrix ${\varvec{D}}$ defined as the diagonal matrix with ${d}_{11},\dots ,{d}_{NN}$ on the diagonal. We use ${\varvec{D}}$ to define generalized eigenvectors ${{\varvec{v}}}_{k}={\left({v}_{k1},\dots ,{v}_{kn}\right)}^{T}$ of ${\varvec{G}}$ with corresponding generalized eigenvalues ${\lambda }_{k}$ , and ${\lambda }_{1}\ge {\lambda }_{2}\ge {\lambda }_{3}\ge \cdots$:

1 $$G{\varvec{v}}_{k} = \lambda_{k} D{\varvec{v}}_{k}$$

Similar to PCA on non-centered or unnormalized data, the first generalized eigenvector of ${\varvec{D}}$ and ${\varvec{G}}$ simply represents the average pattern of all SNPs (see Fig. 1). Therefore, we start from the second generalized eigenvector and define the $k$th component of SUGIBS to be the $k+1$th generalized eigenvector of ${\varvec{G}}$ and ${\varvec{D}}$, ${{\varvec{v}}}_{k+1}$.

By multiplying ${{\varvec{D}}}^{-\frac{1}{2}}$ on both sides of Eq. (1), we obtain:

2 $${\varvec{D}}^{{ - \frac{1}{2}}} G{\varvec{D}}^{{ - \frac{1}{2}}} {\varvec{D}}^{\frac{1}{2}} {\varvec{v}}_{k} = \lambda_{k + 1} {\varvec{D}}^{{ - \frac{1}{2}}} {\varvec{v}}_{k}$$

Subsequently, we observe that the eigenvector ${{\varvec{v}}}_{k}^{^{\prime}}={{\varvec{D}}}^\frac{1}{2}{{\varvec{v}}}_{k}$ of ${{{\varvec{D}}}^{-\frac{1}{2}}{\varvec{G}}{{\varvec{D}}}^{-\frac{1}{2}}=\frac{1}{M}{\varvec{D}}}^{-\frac{1}{2}}{{{\varvec{X}}}^{T}{\varvec{X}}{\varvec{D}}}^{-\frac{1}{2}}$ can be obtained from the singular value decomposition (SVD) of the matrix $\hat{\user2{X}} = {\varvec{XD}}^{{ - \frac{1}{2}}} = {\varvec{U}}{{\varvec{\Sigma}}}{\varvec{V}}^{\prime T}$, where ${{\varvec{v}}}_{k}^{\boldsymbol{^{\prime}}}$ is also the $i$th right singular vector with singular value ${\sigma }_{k}=\sqrt{{M\lambda }_{k+1}}$, ${\varvec{\Sigma}}$ is a $N\times N$ diagonal matrix, ${\varvec{U}}$ is a $M\times N$ matrix with all the left singular vectors and ${\varvec{V}}\boldsymbol{^{\prime}}$ is a $N\times N$ matrix with all the right singular vectors.

Denoting ${{\varvec{U}}}_{k}=\left\{{{\varvec{u}}}_{2},\dots ,{{\varvec{u}}}_{k+1}\right\}$ and ${\boldsymbol{\Sigma }}_{k}=diag\left\{{\sigma }_{2},\dots ,{\sigma }_{k+1}\right\}$, the corresponding left singular vectors and the singular values of the first $k$ SUGIBS components ${{\varvec{V}}}_{k}={{\varvec{D}}}^{-\frac{1}{2}}{{\varvec{V}}}_{k}^{^{\prime}}={{\varvec{D}}}^{-\frac{1}{2}}\left\{{{\varvec{v}}}_{2},\dots ,{{\varvec{v}}}_{k+1}\right\}$, we have the following equation:

3 $${\varvec{V}}_{k} = {\varvec{D}}^{{ - \frac{1}{2}}} {\varvec{V}}_{k}^{\prime } = {\varvec{D}}^{ - 1} {\varvec{S}}_{k} = {\varvec{D}}^{ - 1} {\varvec{X}}^{T} {\varvec{L}}_{k} = {\varvec{D}}^{ - 1} {\varvec{X}}^{T} {\varvec{U}}_{k} {{\varvec{\Sigma}}}_{{\text{k}}}^{ - 1}$$

Thus, we denote ${{\varvec{L}}}_{k}={{\varvec{U}}}_{k}{{\varvec{\Sigma}}}_{\mathrm{k}}^{-1}$ as the SUGIBS loading matrix for the first $k$ SUGIBS components and ${{{\varvec{S}}}_{k}={\varvec{X}}}^{T}{{\varvec{U}}}_{k}{{\varvec{\Sigma}}}_{\mathrm{k}}^{-1}$ as the unnormalized SUGIBS score matrix.

### SUGIBS dataset projection

Given the SUGIBS loadings ${{\varvec{L}}}_{k}$ from a reference dataset with $N$ individuals and $M$ SNPs and given a new dataset with $\stackrel{\sim }{N}$ individuals and the same set of SNPs as the reference sample, we denote the unnormalized genotype matrix of the new dataset as $\stackrel{\sim }{{\varvec{X}}}$. We then define the reference degree ${\stackrel{\sim }{d}}_{ii}={\sum }_{j}^{N}{\stackrel{\sim }{w}}_{ij}$, where ${\stackrel{\sim }{w}}_{ij}$ is denoted as the IBS similarity between the $i$th individual in the target dataset and the $j$th individual in the reference dataset. The reference similarity degree matrix $\stackrel{\sim }{{\varvec{D}}}$ of the new dataset is a diagonal matrix with ${\stackrel{\sim }{d}}_{11},\dots ,{\stackrel{\sim }{d}}_{\stackrel{\sim }{N}\stackrel{\sim }{N}}$ on the diagonal. For the first $k$ SUGIBS components, the projected score matrix of the target dataset is then obtained as:

4 $$\tilde{\user2{V}}_{k} = \tilde{\user2{D}}^{ - 1} \tilde{\user2{S}}_{k} = \tilde{\user2{D}}^{ - 1} \tilde{\user2{X}}^{T} {\varvec{L}}_{k} = \tilde{\user2{D}}^{ - 1} \tilde{\user2{X}}^{T} {\varvec{U}}_{k} {{\varvec{\Sigma}}}_{{\text{k}}}^{ - 1}$$

In Eq. (4), the reference similarity degree matrix $\stackrel{\sim }{{\varvec{D}}}$ acts as a normalization term correcting the missing genotypes and errors in the samples to be projected. As an example, consider a rare SNP with major allele *A* and minor allele *G*, and an individual with true genotype *AA* that is wrongfully coded as *GG* for that particular SNP. Since the major genotype in the reference data of this SNP is *AA*, the number of shared alleles of this SNP between this individual to the majority of individuals in the reference dataset would reduce from 2 to 0. The unnormalized genotype coding of this person also changes from 1 to − 1. Thus, the influence of such a genotyping error on the unnormalized SUGIBS score matrix ${\stackrel{\sim }{{\varvec{S}}}}_{k}$ and the reference similarity degree matrix $\stackrel{\sim }{{\varvec{D}}}$ are along the same direction so that the final SUGIBS scores are corrected by ${\stackrel{\sim }{{\varvec{D}}}}^{-1}$. Other typical batch artefact errors and missing genotypes in the new dataset are corrected for in a similar way and, most interestingly, this correction is provided on an individual by individual basis.

The following is a description of the data used for each of the three experiments. An overview of different data filtering steps for each of the experiments is provided in Supplementary Table S2.

### Experiment 1, individual outlier robustness

The basic dataset to investigate robustness against individual outliers in a reference dataset consists of the individuals from the CEU population (111 individuals) and the TSI population (102 individuals) from the HapMap 3 dataset^3^, after excluding non-founders. Individuals with more than 10% missing genotypes were removed. Related individuals were identified using KING^43^ with threshold 0.044, which is the threshold for 3rd degree relatives, after which one of each pair of related individuals was randomly removed. We randomly selected one individual as outlier from four other populations (CHB, MEX, GIH, and YRI). These individuals specifically are NA18798 (CHB), NA19740 (MEX), NA21124 (GIH), and NA19262 (YRI). After removing the monomorphic SNPs in each of these three datasets, we built SUGIBS, MDS, UPCA and PCA spaces using 892,338 autosomal SNPs remaining in all three datasets. We intentionally did not perform either minor allele frequency (MAF) filtering or HWE filtering on the SNPs since many rare SNPs and SNPs violating HWE are due to the outliers and were therefore not checked for during the testing for robustness.

### Experiment 2, simulated laboratory artefacts

We used the 1,000 Genomes Project dataset (2,504 unrelated individuals from 26 populations) as the reference dataset to infer ancestry spaces. We used the HGDP dataset that analyzed genomic data from 1,043 individuals from around the world as the dataset to project. For both 1KGP and HGDP dataset, individuals with more than 10% missing genotypes were removed. For the 1KGP only, related individuals were identified using KING^43^ with threshold 0.044, after which one of each pair of related individuals was randomly removed. First, we remapped the HGDP dataset from the NCBI36 (hg18) assembly to the GRCh37 (hg19) assembly using the NCBI Genome Remapping Service. We further performed a LD pruning with a window size of 50, a moving step of 5 and a threshold ${r}^{2}>0.2$ for several times until no more SNPS were excluded^12^. LD pruning is a common practice when using PCA. Therefore, we followed this additional step to make the results based on PCA, UPCA and SUGIBS comparable for this particular experiment. Doing so, we selected 154,199 autosomal SNPs to construct the ancestry spaces. We then extracted the first eight ancestry components from the reference dataset. After extracting the same set of SNPs in the HGDP dataset, we took care to ensure that the alternate alleles were the same as in the reference dataset.

Since PLINK binary file format stores the genotypes of four consecutive individuals in a single byte, we assigned one of every two “bytes” (four individuals) into dataset A (the dataset to modify) and the other individuals into dataset B (the dataset to remain unchanged) of the HGDP dataset. This resulted in 523 individuals for dataset A and 520 individuals for dataset B. In order to simulate laboratory artefacts, we randomly masked 5% genotype calls as missing and changed 5% genotype calls (e.g., from AA to Aa or aa) of the rare SNPs (MAF < 0.05) in dataset A. Random genotype masking and changing were also performed on the “byte” level, i.e. four individuals at a time. For both genotyping masking and changing, we generated 100 datasets to project on the 1KGP reference ancestry space. Subsequently, as input to Fig. 3, we calculated the root-mean-square deviations (RMSD) between the scores of the top eight ancestry axes generated using the original genotypes and the modified genotypes in dataset A and further normalized them by the range of the axes generated using the original genotypes so that normalized root-mean-square deviations (NRMSD) across methods are comparable.

### Experiment 3A, simulated admixed population

Our admixture simulations were adapted from the section “Simulation Framework” in Galinsky et al.^12^. 100 times we similated data at 3,200 random independent SNPs for 200 individuals. For a given SNP $i$, the ancestral allele frequency ${p}_{i}$ was sampled from a $Uniform\left(\mathrm{0.1,0.9}\right)$ distribution. Population allele frequencies were generated by simulating random drift in two populations of fixed effective size ${N}_{e}$ for $\tau$ generations as ${p}_{i1}$ and ${p}_{i2}$, whose initial values were set to ${p}_{i}$. In each generation, the number of alternate alleles ${z}_{i1}$ and ${z}_{i2}$ were sampled from two binomial distributions with ${2N}_{e}$ number of trials and ${p}_{i1}$ and ${p}_{i2}$ success probabilities. The population allele frequencies were then updated by ${p}_{i1}=\frac{{z}_{i1}}{2{N}_{e}}$ and ${p}_{i2}=\frac{{z}_{i2}}{2{N}_{e}}$. For all simulations, population allele frequency simulations were run for 20 generations and the effective population size ${N}_{e}$ was calculated for a target $F_{st}$ by $F_{st} = - {\log}\left( {1 - \frac{\tau }{{2N_{e} }}} \right)$^44^. This was done for ${F}_{st}=\{0.001, 0.005, 0.01, 0.05, 0.1\}$, ${N}_{e}\approx \{10k,2k,1k,200, 100\}$ with $\tau =20$.

The ancestry proportions ${\alpha }_{j}$ were sampled from a $beta\left(0.5, 0.5\right)$ distribution so that the proportion from each ancestry is 50% on average. For a given individual $j$ with ancestry proportion of ${\alpha }_{j}$ from Population one and $\left(1-{\alpha }_{j}\right)$ from population two, the individual allele frequency for SNP $i$ was ${p}_{i}^{j}={\alpha }_{j}{p}_{i1}+\left(1-{\alpha }_{j}\right){p}_{i2}$ and the genotype was sampled from a binomial distribution with 2 trials and ${p}_{i}^{j}$ success probability.

### Experiment 3B, simulated GWAS

Our GWAS simulation is similar to the one carried out in Price et al.^14^. To simulate a case–control GWAS, we generated 1,000,000 SNPs for 1,000 individuals from a population admixed from two ancestries. The case–control status was simulated using a disease risk proportional to ${r}^{\alpha }$, based on an ancestral risk of $r=3$. We generated three categories of SNPs (random, differentiating and causal) to compare the performance of PCA, MDS, and SUGIBS in correcting for population stratification. For the first category (random SNPs with no association to the disease), we generated the SNPs by simulating random drift with a certain ${F}_{st}$ divergence. For the second category (differentiated SNPs with no association), we assumed population allele frequencies of 0.8 for ancestry one and 0.2 for ancestry two. For the third category (causal SNPs), we generated SNPs by combining a multiplicative disease risk model while simulating the random drift with the same ${F}_{st}$ as the random SNPs.

We simulated the case–control status according to Price et al.^7^. For individuals with an ancestry proportion of $\alpha$ from population one and $\left(1-\alpha \right)$ from population two, the case–control status was simulated with the probability of disease equal to $\frac{\mathrm{log}\left(r\right){r}^{a}}{2(r-1)}$ , which ensures an average value of 0.5 across all the values of $\alpha$^7^. For the case individuals, the population allele frequencies ${p}_{i1}$ and ${p}_{i2}$ of the causal SNP $i$ were further updated to ${p}_{i1}^{*}=\frac{R{p}_{i1}}{1-{p}_{i1}+R{p}_{i1}}$ and ${p}_{i2}^{*}=\frac{R{p}_{i2}}{1-{p}_{i2}+R{p}_{i2}}$ with a relative risk of $R=3$, respectively.

### PSU cohort and 3D facial images

Study participants in the PSU cohort were collected with informed consent as part of several studies based at The Pennsylvania State University and sampled in the following locations: State College, PA (IRB #44929 & #4320); New York, NY (#45727); Urbana-Champaign, IL (#13103); Cape Verde; Dublin, Ireland; Rome, Italy; Warsaw, Poland, and Porto, Portugal (#32341); and Twinsburg, OH (#2503). The individuals were genotyped on the 23andMe v3 and v4 arrays (23andMe, Mountainview, CA) and Illumina HumanHp200v1 BeadChip (Illumina Inc., San Diego, CA) platforms. After filtering out SNPs with more than 10% missing genotypes, the intersection of these two arrays compromised of approximately 600 K SNPs. We removed individuals with misclassified sex information, missing covariate data, and those with more than 10% missing genotypes. Relatives were identified as pairs of individuals using a KING^43^ with threshold (0.044, which is the threshold for 3^rd^ degree relatives), after which one of each pair was randomly removed, resulting in a set of 2,882 individuals. Genotypes were imputed to the 1,000 Genomes Project Phase 3 reference panel, using SHAPEIT2 for prephasing of haplotypes and imputed using the Sanger Imputation Server PBWT pipeline.

3D facial images were taken using the 3dMDface (3dMD, Atlanta, GA) and the Vectra H1 (Canfield, Parsippany, NJ) imaging systems. Height and weight were measured using an Accustat stadiometer (Genentech, San Francisco, CA), a clinical scale (Tanita, Arlington Heights, IL), or by self-report. 3D facial images were imported into matlab 2016b in .obj wavefront format to perform spatially dense registration (MeshMonk^45^). This resulted in homologous spatially dense configurations of 7,160 quasi-landmarks per facial image. In other words, each 3D image is represented with the same amount of 3D points that are in full one-to-one correspondence with all other images. Reflected images were created by changing the sign of the x-coordinate of the original mapped images. Both the original and the reflected remapped faces were then superimposed following a generalized Procrustes superimposition to eliminate differences in orientation, position and scale^46^. Symmetrized images were created by averaging the original and the reflected images.

### Constructing ancestry-derived average 3D faces

In total, we had 69,194 overlapping autosomal SNPs that were present in the 1KGP dataset, the PSU cohort and the ancient-DNA profiles following a MAF filtering of 1%, HWE filtering with p-value threshold 1e-6, and LD pruning: window size, 50 step size, 5 and LD threshold of 0.2. Based on this selection, we constructed 25 SUGIBS ancestry components using the 1KGP dataset, which is theoretically sufficient to separate 26 populations, from the 1KGP reference dataset. Subsequently, we projected the 2,882 individuals from the PSU cohort and the eight ancient-DNA profiles onto the 1KGP ancestry components using the same 69,194 overlapping autosomal SNPs. Then, we fitted a partial least-squares regression (PLSR) model (plsregress function in matlab 2016b) using the superimposed and symmetrized 3D facial images with 7,160 quasi-landmarks collected in the PSU cohort as the response variables, and the first eight projected SUGIBS scores of the PSU cohort together with four covariates [age (years), sex (1 = male; 2 = female), height (cm) and weight (kg)] as the explanatory or predictor variables.

Given specific ancestry scores on the ancestry components of the 1KGP ancestry space, together with age, height, weight and sex, the PLSR model was able to generate an ancestry-derived average face as response variable. To illustrate the ancestry components in Fig. 5, we simply varied a single score along each ancestry component separately, while keeping the scores on the other ancestry components fixed and equal to zero with following values for age = 30 years, height = 165 cm, weight = 75 kg and sex = 1.5 (reflecting a genderless face). For example, for the first axis in Fig. 5(a), the face along the positive direction of this axis was constructed using X = [30 1.5 165 75 mean (axis1_1kgp_ ) + 3*std (axis1_1kgp_) 0 0 0 0 0 0 0] and the face along the negative direction of this axis was constructed using X = [30 1.5 165 75 mean (axis1_1kgp_) − 3*std (axis1_1kgp_) 0 0 0 0 0 0 0]. The projected ancestry scores of the eight ancient-DNA profiles were used together with the genome-derived sex values, age = 30 years, height = 165 cm, weight = 75 kg as predictors in the PLSR model to construct the ancestry average 3D faces of the respective ancient-DNA profiles.

### Ethics statement

Institutional review board (IRB) approval was obtained at each recruitment site and all participants gave their written informed consent prior to participation; for children, written consent was obtained from a parent or legal guardian. For the PSU cohort, the following local ethics approvals were obtained: State College, PA (IRB #44929 and #4320 New York, NY (IRB #45727); Urbana-Champaign, IL (IRB #13103); Dublin, Ireland; Rome, Italy; Warsaw, Poland; and Porto, Portugal (IRB #32341); and Twinsburg, OH (IRB #2503).

### Consent for publication

All methods were carried out in accordance with relevant guidelines and regulations.

## Supplementary information

Supplementary information.

## Data Availability

The data used to develop, validate and test SUGIBS in this work originates solely from public open-source projects, including the HapMap 3 project, 1,000 Genome project and the HGDP dataset. For access to this data, we refer to their respective webpages as indicated under the URL section. An implementation of SUGIBS is freely available (see URLs below). This comprises a matlab toolbox, referred to as SNPLIB, and contains implementations of all the methods and including the simulations used in this work. The data used to apply SUGIBS in the context of facial shape variations was based on participants comprising the Penn State University dataset (PSU cohort). However, these were not collected with broad data sharing consent. Given the highly identifiable nature of both facial and genomic information and unresolved issues regarding risks to participants of inherent reidentification, participants were not consented for inclusion in repositories or the posting of individual data. Broad data sharing of the PSU cohort would thus be both a legal and ethical violation of the informed consent document signed by the researchers and participants. This restriction is not because of any personal or commercial interests. However, on the webpage of SNPLIB (see URLs below), we do provide the resulting PLSR model and other necessary data (without the need to access the raw images), along with demo scripts, to replicate the ancestry facial images obtained in this work. HapMap 3 Data: https://www.genome.gov/10001688/international-hapmap-project/. 1,000 Genome Project: https://www.internationalgenome.org/. HGDP dataset: https://www.cephb.fr/hgdp/. SNPLIB: https://github.com/jiarui-li/SNPLIB. MeshMonk: https://github.com/TheWebMonks/meshmonk. NCBI Genome Remapping Service: https://www.ncbi.nlm.nih.gov/genome/tools/remap.
