# Supplementary material for: Robust genome-wide ancestry inference for heterogeneous datasets: illustrated using the 1,000 genome project with 3D facial images
Source: Sci Rep. 2020 Jul 16;10:11850. doi: 10.1038/s41598-020-68259-w (PMC7367291; doi:10.1038/s41598-020-68259-w)
Supplement: Supplementary file 1 — Supplementary information. [file 41598_2020_68259_MOESM1_ESM.pdf]

Robust Genome-Wide Ancestry Inference for Heterogeneous Datasets: illustrated using the 1000 Genome Project with 3D Facial Images

Jiarui Li, Tomás González Zarzar, Julie D. White, Karlijne Indencleef, Hanne Hoskens, Harry Matthews, Nele Nauwelaers, Arslan Zaidi, Ryan J. Eller, Noah Herrick, Torsten Günther, Emma M. Svensson, Mattias Jakobsson, Susan Walsh, Kristel Van Steen, Mark D. Shriver, Peter Claes

Supplementary Materials

Tables

Supplementary Table S1: Information and reference on eight ancient DNA profiles.

Supplementary Table S2: SNP filtering and LD-pruning information.

Figures

Supplementary Figure S1: Top eight SUGIBS axes of 1KGP (African subpopulations highlighted) and projections of the PSU cohort.

Supplementary Figure S2: Top eight SUGIBS axes of 1KGP (Admixed American subpopulations highlighted) and projections of the PSU cohort.

Supplementary Figure S3: Top eight SUGIBS axes of 1KGP (East Asian subpopulations highlighted) and projections of the PSU cohort.

Supplementary Figure S4: Top eight SUGIBS axes of 1KGP (European subpopulations highlighted) and projections of the PSU cohort.

Supplementary Figure S5: Top eight SUGIBS axes of 1KGP (South Asian subpopulations highlighted) and projections of the PSU cohort.

Notes

Supplementary Note S1: Determination of the number of relevant SUGIBS components

Supplementary Note S2: The use and pitfalls of ancestry facial predictions

Supplementary Table S1:

Table S1: Information and reference on eight ancient DNA profiles.

Individual ID	Geography	Time (years cal BP)	Reference
Anzick-1	North America	12,707–12,556	Rasmussen et al. (2014)[1]
baa001	Southern Africa	1,986–1,831	Schlebush et al. (2017)[2]
Kotias	Caucasus	9,529–9,895	Jones et al.(2015)[3]
LBK	Europe	7,450-6,750	Lazaridis et al. (2014)[4]
Loschbour	Europe	8,170-7,940	Lazaridis et al. (2014)[4]
ne1	Europe	7,020–7,290	Gamba et al. (2014)[5]
SF12	Northern Europe	9,033–8,757	Günther et al. (2018)[6]
Ust_Ishim	Western Siberia	46,880–43,210	Fu et al. (2014)[7]

Supplementary Table S2:

Table S2: SNP filtering and LD-pruning information. 1KGP, 1000 genome project. PSU, The Pennsylvania State University, MAF, minor allele frequency, HWE, Hardy-Weinberg Equilibrium, LD, Linkage Disequilibrium, SNP, single nucleotide polymorphism, HGDP, Human Genome Diversity Project, IBS Identity-by-state, KING[8] (0.044 is the threshold for 3rd degree of relatives)

<table>
<tr><th>Paper Context</th><th>Dataset</th><th>Experiment</th><th>Missing Genotypes</th><th>Related Individuals</th><th>MAF-Filtering</th><th>HWE-Filtering</th><th>LD-Pruning</th><th>#SNPs</th><th colspan="2">COMMENTS</th></tr>
<tr><td>Outlier robustness</td><td>HapMap 3</td><td>Figure 1</td><td>>10%</td><td>KING>.044</td><td>No</td><td>No</td><td>No</td><td>892,338</td><td rowspan="2">We did not perform either minor allele frequency (MAF) filtering or HWE filtering on the SNPs since many rare SNPs and SNPs violating HWE are due to the outliers and therefore useful in testing for robustness.</td><td></td></tr>
<tr><td>Laboratory artefacts</td><td>HGDP & 1KGP</td><td>Figure 2, 3</td><td>>10%</td><td>KING>.044 on 1KGP

No relative removal on HGDP</td><td>No</td><td>No</td><td>window size:50 step size:5 threshold:0.2</td><td>154,199</td><td>We perform the LD-pruning here mainly to reduce the number of SNPs for computational reasons only, because the simulations run for 100 times.</td></tr>
<tr><td>Revealing population structure</td><td>Simulated Admixture</td><td>Figure 4</td><td>No</td><td>No</td><td>No</td><td>No</td><td>No</td><td>3,200</td><td>Galinsky et al.[9]</td><td></td></tr>
<tr><td>Adjusting population structure</td><td>Simulated GWAS</td><td>Table 1</td><td>No</td><td>No</td><td>No</td><td>No</td><td>No</td><td>1,000,000</td><td>Price et al.[10]</td><td></td></tr>
<tr><td>Imaging 3D Facial Variations</td><td>Ancient DNA, PSU cohort, and 1KGP</td><td>Figure 5,6, S1-S5</td><td>>10%</td><td>KING>.044</td><td>.01</td><td>1.00E-06</td><td>window size:50 step size:5 threshold:0.2</td><td>69,194</td><td></td><td></td></tr>
</table>

Supplementary Figure S1:

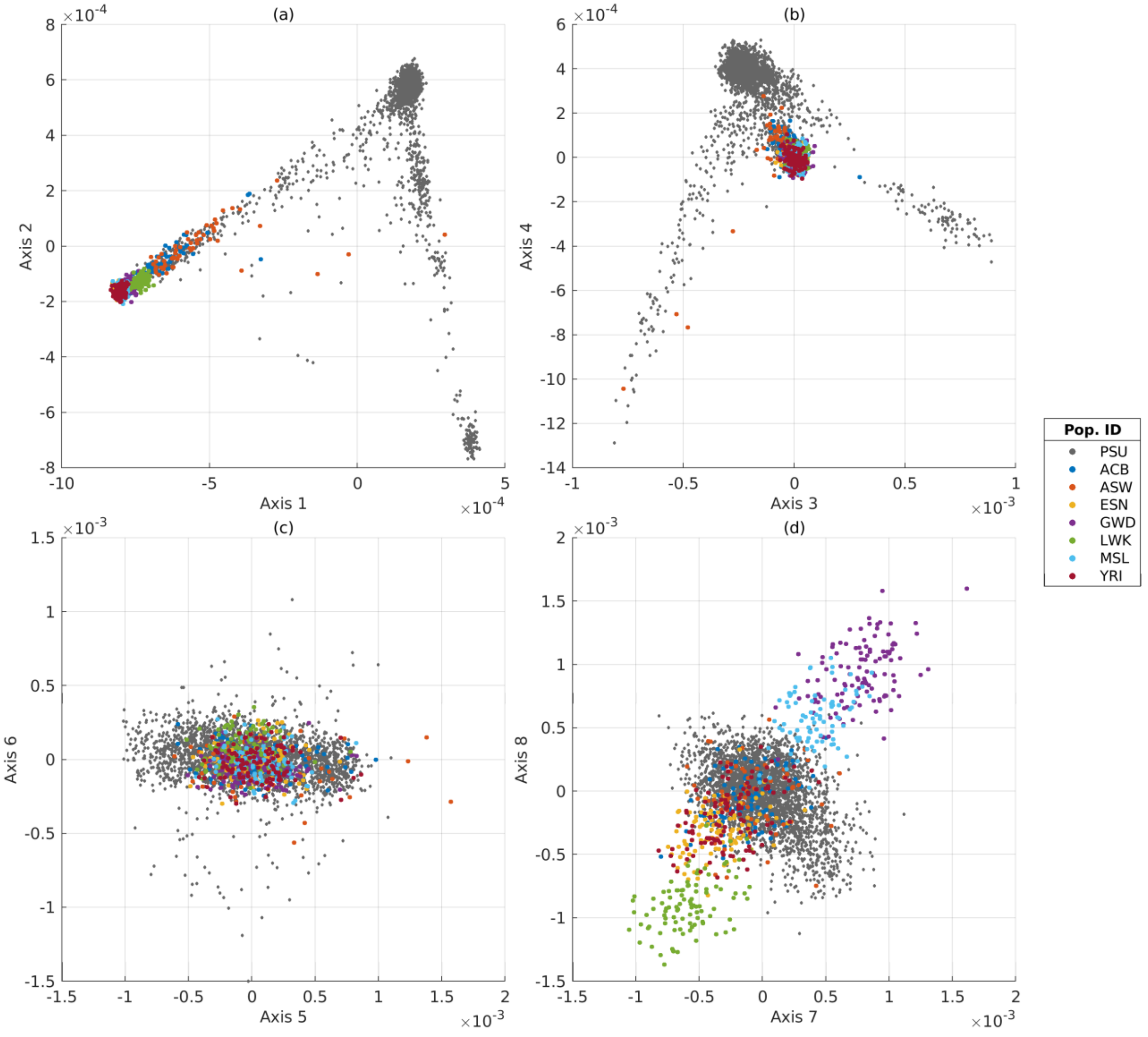

Figure S1: Top eight SUGIBS axes of 1KGP and projections of the PSU cohort. Populations of the African super population in the 1KGP are coloured. The projected PSU cohort are represented by grey dots. ACB, African Caribbeans in Barbados, ASW, Americans of African Ancestry in SW USA, ESN, Esan in Nigeria, GWD, Gambian in Western Divisions in the Gambia, LWK, Luhya in Webuye Kenya, MSL, Mende in Sierra Leone, YRI, Yoruba in Igadan Nigeria. (a) SUGIBS axis 1 (Horizontal) and axis 2 (Vertical). (b) SUGIBS axis 3 (Horizontal) and axis 4 (Vertical). (c) SUGIBS axis 5 (Horizontal) and axis 6 (Vertical). (d) SUGIBS axis 7 (Horizontal) and axis 8 (Vertical).

Supplementary Figure S2:

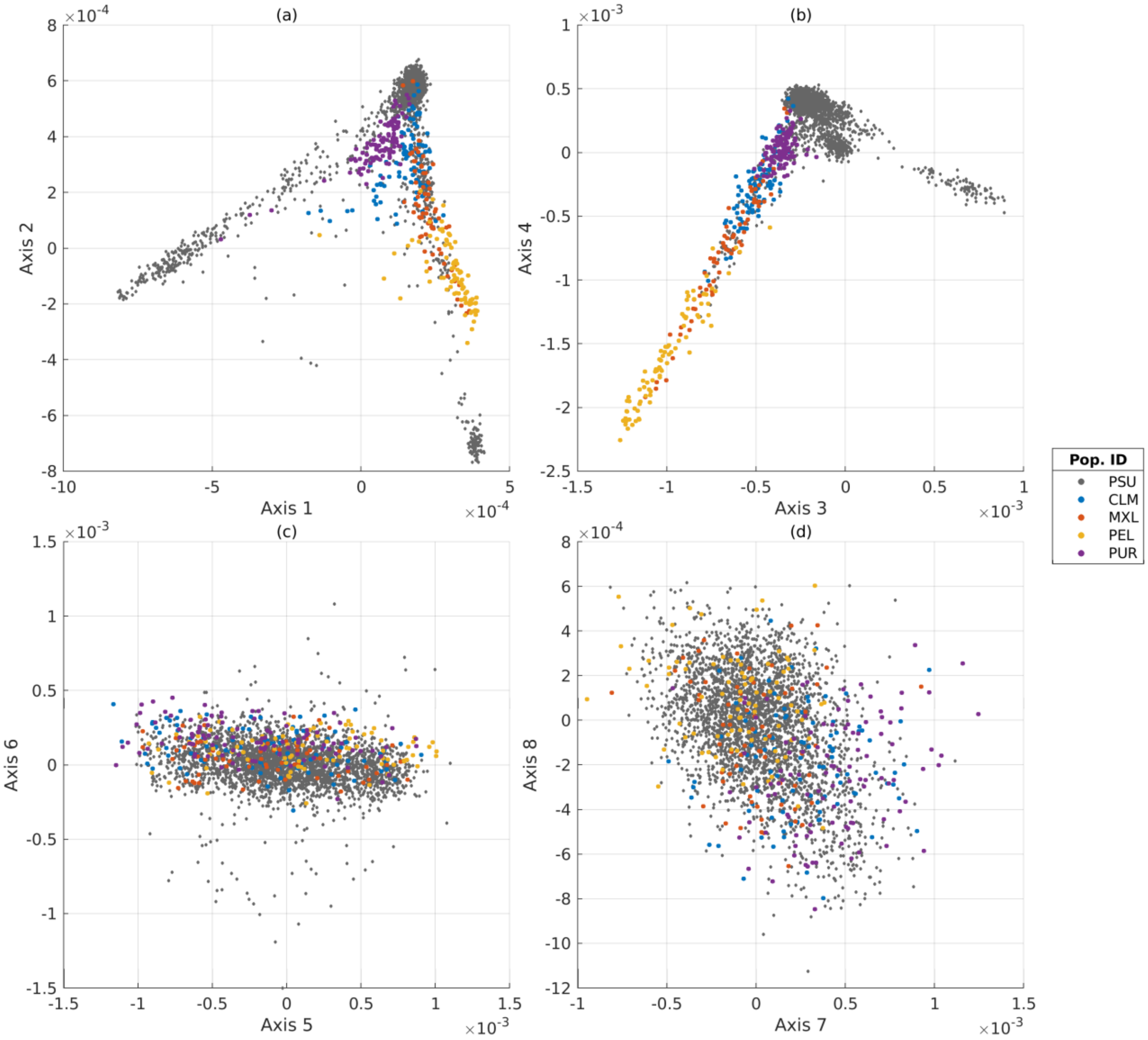

Figure S2: Top eight SUGIBS axes of 1KGP and projections of the PSU cohort. Populations of the admixed American super population in the 1KGP are coloured. The projected PSU cohort are represented by grey dots. CLM, Colombians from Medellin Columbia, MXL, Mexican ancestry from Los Angeles USA, PEL, Peruvians from Lima, Peru, PUR, Puerto Ricans from Puerto Rico. (a) SUGIBS axis 1 (Horizontal) and axis 2 (Vertical). (b) SUGIBS axis 3 (Horizontal) and axis 4 (Vertical). (c) SUGIBS axis 5 (Horizontal) and axis 6 (Vertical). (d) SUGIBS axis 7 (Horizontal) and axis 8 (Vertical).

Supplementary Figure S3:

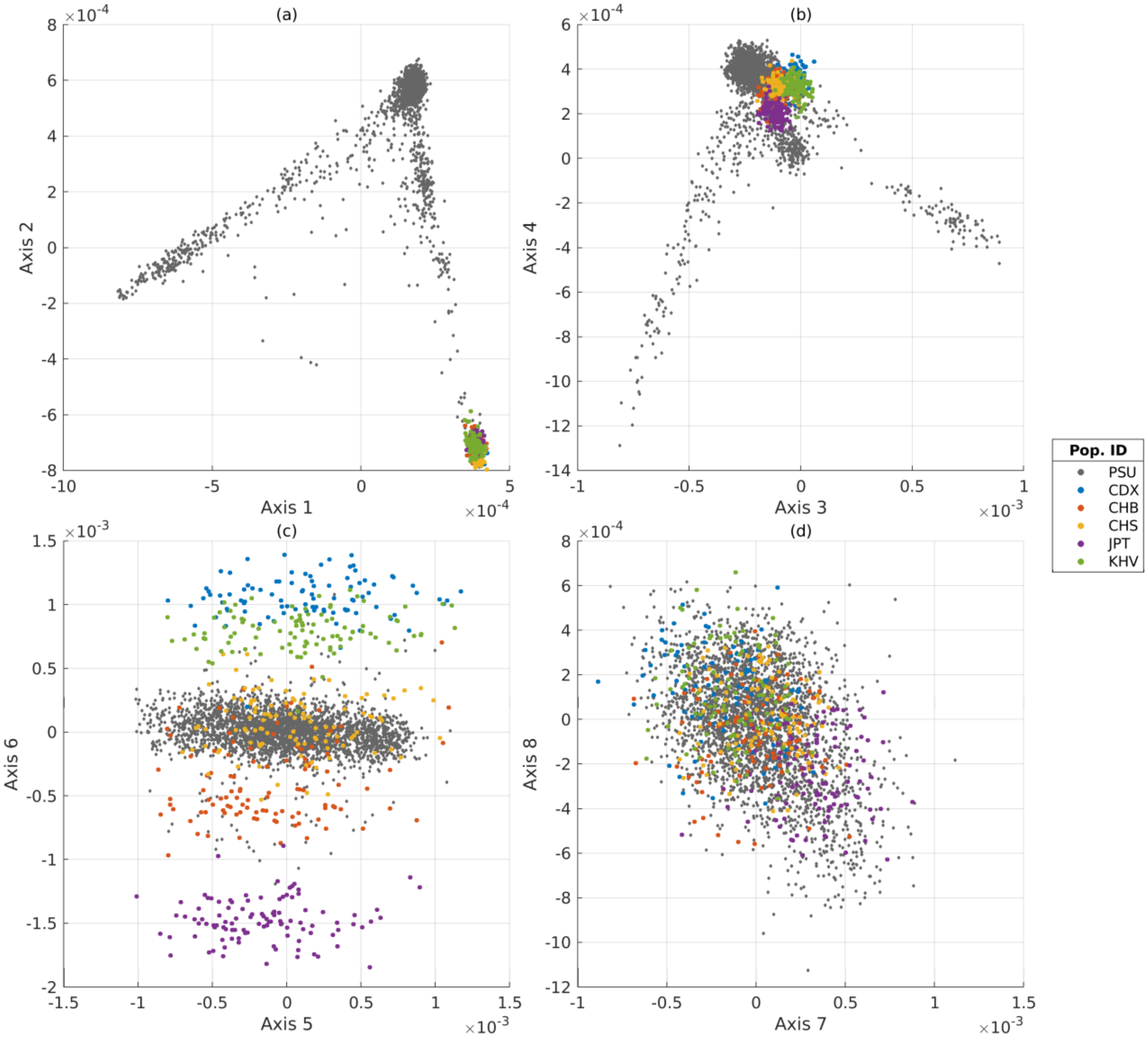

Figure S3: Top eight SUGIBS axes of 1KGP and projections of the PSU cohort. Populations of the East Asian super population in the 1KGP are coloured. The projected PSU cohort are represented by grey dots. CDX, Chinese Dai in Xishuangbanna China, CHB, Han Chinese in Beijing China, CHS, Southern Han Chinese, JPT, Japanese in Tokyo Japan, KHV, Kinh in Ho Chi Minh City Vietnam. (a) SUGIBS axis 1 (Horizontal) and axis 2 (Vertical). (b) SUGIBS axis 3 (Horizontal) and axis 4 (Vertical). (c) SUGIBS axis 5 (Horizontal) and axis 6 (Vertical). (d) SUGIBS axis 7 (Horizontal) and axis 8 (Vertical).

Supplementary Figure S4:

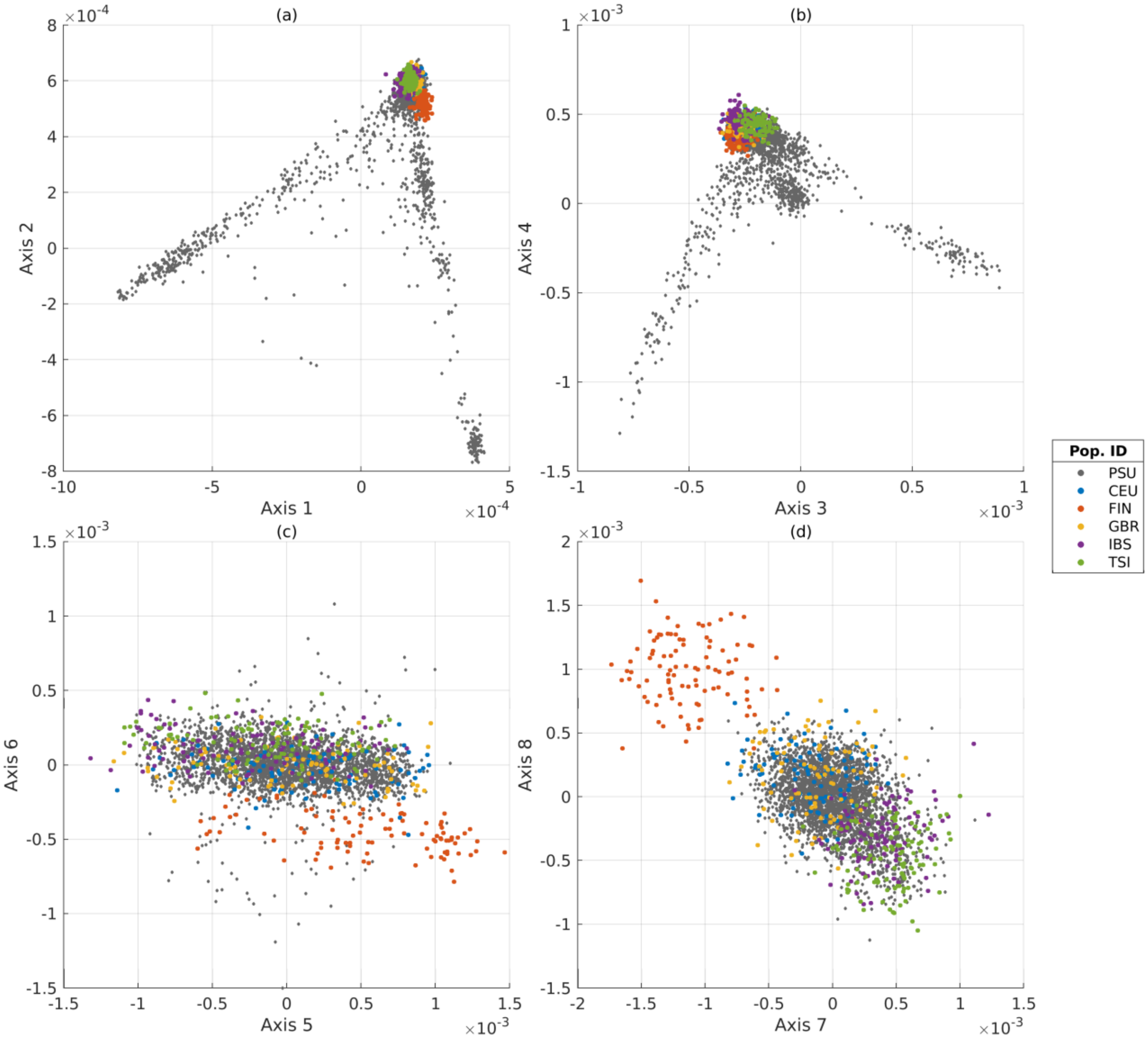

Figure S4: Top eight SUGIBS axes of 1KGP and projections of the PSU cohort. Populations of the European super population in the 1KGP are coloured. The projected PSU cohort are represented by grey dots. CEU, Utah Residents (CEPH) with Northern and Western European ancestry, FIN, Finnish in Finland, GBR, British in England and Scotland, IBS, Iberian population in Spain, TSI, Toscani in Italia. (a) SUGIBS axis 1 (Horizontal) and axis 2 (Vertical). (b) SUGIBS axis 3 (Horizontal) and axis 4 (Vertical). (c) SUGIBS axis 5 (Horizontal) and axis 6 (Vertical). (d) SUGIBS axis 7 (Horizontal) and axis 8 (Vertical).

Supplementary Figure S5:

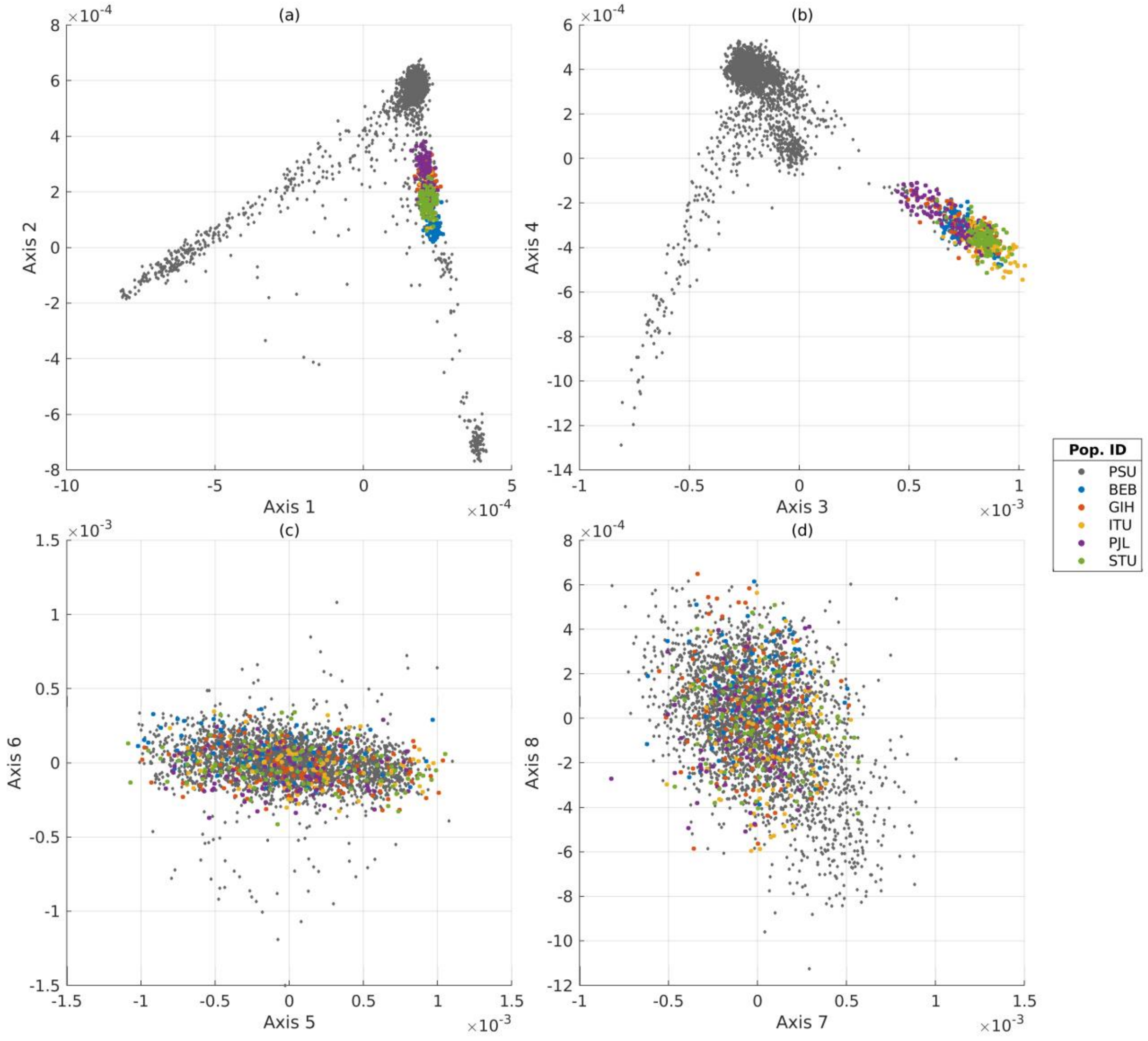

Figure S5: Top eight SUGIBS axes of 1KGP and projections of the PSU cohort. Populations of the South Asian super population in the 1KGP are coloured. The projected PSU cohort are represented by grey dots. BEB, Bengali from Bangladesh, GIH, Gujarati Indian from Houston Texas, ITU, Indian Telugu from the UK, PJL, Punjabi from Lahore Pakistan, STU, Sri Lankan Tamil from the UK. (a) SUGIBS axis 1 (Horizontal) and axis 2 (Vertical). (b) SUGIBS axis 3 (Horizontal) and axis 4 (Vertical). (c) SUGIBS axis 5 (Horizontal) and axis 6 (Vertical). (d) SUGIBS axis 7 (Horizontal) and axis 8 (Vertical).

Supplementary Note S1: Determination of the number of relevant SUGIBS components

A key question for any lower dimensional embedding of data into a latent-space is the determination of the number of relevant latent components. In previous work [11], we used PCA to obtain lower dimensional facial shape presentations in combination with a technique referred to as Parallel Analysis [12,13]. A Parallel Analysis determines the amount of eigenvalues (and thus the number of principal components (PCs)) from the observed data that are significantly different from eigenvalues computed from permuted versions of the original data. By running multiple permutations, a null distribution of noisy eigenvalues is obtained, against which significance of the original eigenvalues can be tested (whilst taking the properties of the data itself into account). Similar to a Parallel Analysis in PCA[12], our preliminary method or suggestion to select the number of components for SUGIBS is by comparing the spectrum of eigenvalues from an observed potentially heterogeneous dataset (HED) with that of simulated homogeneous datasets (HOD). This is done using the same number of SNPs and samples as in the observed dataset.

For the HODs, we generate the genotypes of each SNP independently according to the allele frequency calculated from the observed data. This implies that each SNP is in HWE but is not in LD with any other SNP. For each simulated HOD and the HED, we calculate the eigenvalues of $\boldsymbol{D}^{-\frac{1}{2}}\boldsymbol{G}\boldsymbol{D}^{-\frac{1}{2}}$, where the unnormalized genomic relationship matrix is defined as $\boldsymbol{G}$ and $\boldsymbol{D}$ is a similarity degree matrix defined by the IBS similarity. By comparing the eigenvalues of the HEDs with the eigenvalues from the simulated HODs, an indication whether the observed dataset deviates from a single homogeneous population is provided. However, we observed that the LD between the SNPs in a sample does affect the sloop of the eigenvalue spectrum. To illustrate this, we simulated three datasets each with 10,000 SNPs and 1,000 samples assuming homogeneity, but with different levels of LD between SNPs (no LD, $r^2 \leq 0.2$ and $r^2 \leq 0.8$). The results in Figure S3 show that the higher the LD level, the steeper the eigenvalue spectrum becomes. In other words, the first eigenvalues explain more of the total variance due to correlation in the data, which is expected given the increased levels of LD.

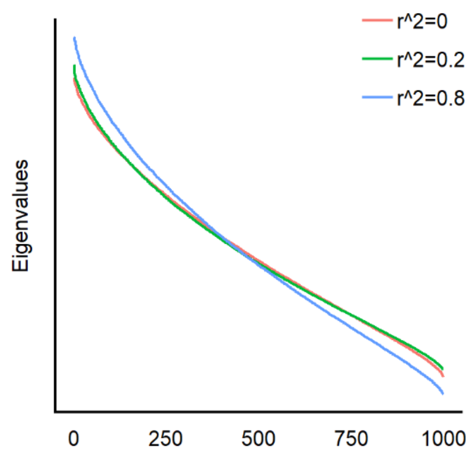

Figure S3: Spectrum in descending order in function of LD level. Y-axis represents the values of eigenvalues.

In order to adjust for the different slopes of the eigenvalue spectrum caused by different levels of LD, we fit a robust regression (robustfit in MATLAB) between the observed eigenvalue spectrum and the simulated eigenvalue spectrum. A robust regression, was chosen since it is not influenced by the first few large eigenvalues, which are expected for highly heterogeneous population samples. In practice, we run the simulation 100 times and robustly fit the observed eigenvalue spectrum with the median of the 100 simulated eigenvalue spectrums. Subsequently, we plot the observed eigenvalue spectrum against the adjusted simulated eigenvalue spectrums.

Results for simulated heterogeneous population samples with an admixture from three, six and nine different ancestries with different levels of F_{st} (0.1, 0.01 and 0.001) are shown in Figure S4. It is observed that the simulated HOD eigenvalue spectrum is consistently lower than the observed HED eigenvalue spectrum, and this for all 30 eigenvalues plotted. Therefore, in contrast to Parallel Analysis, the simulated HOD eigenvalue spectrum could not be used as a direct indicator for the number of significant components, since all the observed eigenvalues remain larger (and thus significant) compared to the simulated ones. However, an indication of the amount of relevant (instead of significant) components that represent admixture is still observed. For larger values of F_{st} (0.1, 0.01), the correct number of relevant components (2 for three ancestries, 5 for six ancestries and 8 for nine ancestries), are visually distinct in magnitude in comparison to the simulated HOD eigenvalue spectrum, and this distinction is larger than the subsequent (non-relevant) components. For lower values of F_{st} (0.001), and an admixture from more than 3 ancestries, this visual distinction is lost.

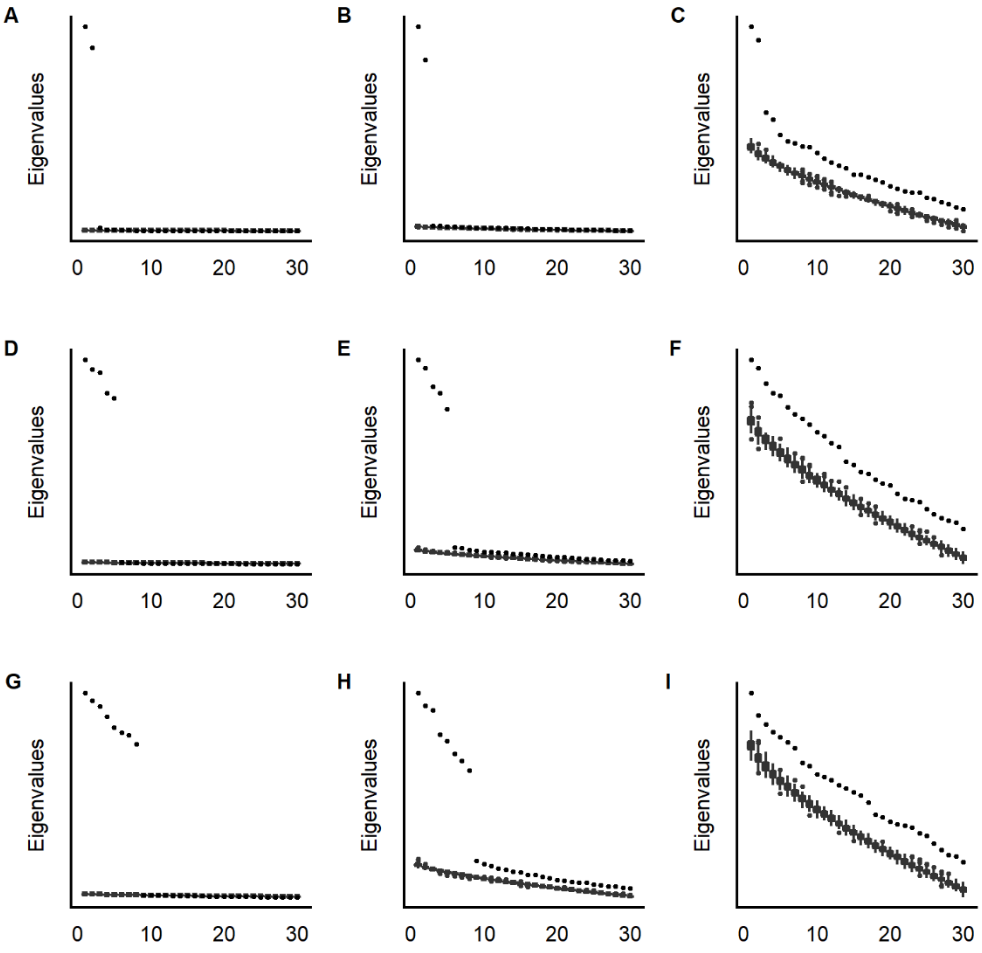

Figure S4: Indicative evaluation of the number of relevant components for simulated heterogeneous population samples. Each simulated heterogeneous population sample is an admixture from A) three ancestries with $F_{st} = 0.1$, B) three ancestries with $F_{st} = 0.01$, C) three ancestries with $F_{st} = 0.001$, D) six ancestries with $F_{st} = 0.1$, E) six ancestries with $F_{st} = 0.01$, F) six ancestries with $F_{st} = 0.001$, G) nine ancestries with $F_{st} = 0.1$, H) nine ancestries with $F_{st} = 0.01$, and I) nine ancestries with $F_{st} = 0.001$. For each simulated admixed as well as homogenous population sample was generated using 1,000 samples with 10,000 SNPs.

Supplementary Note S2: The use and pitfalls of ancestry facial predictions

Ancestry facial predictions have good value in a range of applications. In archeology, ancestry faces reconstructed from ancient DNA profiles, as done in this work, is of strong interest. Generally, for ancient DNA profiles, missing data is abundantly present, making SUGIBS a valuable technique to use. Note that, the ancestry faces are limited to modern facial constructs, due to the contemporary facial data used. However, they can help to bring ancient DNA profiles into the context of present-day populations for which facial images (e.g. open-source facial databases, Google images, etc.) are available but DNA is not. Furthermore, there is a good relationship between the face and the skull[14,15], such that ancestry faces can be used to compare against skeletal remains. In the future, it is of interest to deploy our work on datasets of 3D skeletal craniofacial surfaces extracted from Computer Tomography (CT) or Magnetic Resonance Imaging (MRI). In medicine, and more particularly in oral and maxillofacial surgery, the surgical reconstruction of a patient's face benefits from a proper notion of normal facial shape[16]. In the next five to 20 years, whole genome sequencing will become the standard of care in clinics and a patient-specific ancestry face provides a personalized norm of facial shape towards precision medicine in surgical planning.

In forensics, an ancestry facial prediction circumvents the often legally debated reporting of ancestry proportions of a probe DNA profile in a criminal investigation. In France, for example, DNA phenotyping of externally visible traits is legally allowed, since such traits are considered to be public. However, and in contrast, genomic ancestry proportions, as typically reported in forensic DNA testing, is considered to be private information and cannot be used during criminal investigations. We agree that ancestry proportions are not an externally visible characteristic of an individual. The construction of ancestry proportions is also inherently flawed by labelling the individual into so-called parental populations. Furthermore, such numeric information is hard to interpret and use by a forensic investigator. The reconstruction of an ancestry face on the other hand, avoids needing to explicitly label a DNA profile in function of parental populations and provides a visual feedback to an investigator that is perceptually useful, even in admixed cases. However, a strong limitation is that the ancestry projection and face creation is only as good as the data used to create it. If your background face data doesn't match the ancestry of your test data, then your estimation of the face will remain poor. The challenge in forensics also involves the ability to reconstruct ancestry faces using often limited and contaminated DNA material. Another strong limitation is of ethical concerns that warrants us of the misuse of DNA and facial recognition technology in general beyond the positive implications of solving crime[17].

References:

1. Rasmussen, M. *et al.* The genome of a Late Pleistocene human from a Clovis burial site in western Montana. *Nature* **506**, 225–229 (2014).
2. Schlebusch, C. M. *et al.* Southern African ancient genomes estimate modern human divergence to 350,000 to 260,000 years ago. *Science* **358**, 652–655 (2017).
3. Jones, E. R. *et al.* Upper Palaeolithic genomes reveal deep roots of modern Eurasians. *Nat. Commun.* **6**, 8912 (2015).
4. Lazaridis, I. *et al.* Ancient human genomes suggest three ancestral populations for present-day Europeans. *Nature* **513**, 409–413 (2014).
5. Gamba, C. *et al.* Genome flux and stasis in a five millennium transect of European prehistory. *Nat. Commun.* **5**, 5257 (2014).
6. Günther, T. *et al.* Population genomics of Mesolithic Scandinavia: Investigating early postglacial migration routes and high-latitude adaptation. *PLOS Biol.* **16**, e2003703 (2018).
7. Fu, Q. *et al.* Genome sequence of a 45,000-year-old modern human from western Siberia. *Nature* **514**, 445–449 (2014).
8. Manichaikul, A. *et al.* Robust relationship inference in genome-wide association studies. *Bioinformatics* **26**, 2867–2873 (2010).
9. Galinsky, K. J. *et al.* Fast Principal-Component Analysis Reveals Convergent Evolution of ADH1B in Europe and East Asia. *Am. J. Hum. Genet.* **98**, 456–472 (2016).
10. Price, A. L. *et al.* Principal components analysis corrects for stratification in genome-wide association studies. *Nat. Genet.* **38**, 904–909 (2006).
11. Claes, P. *et al.* Genome-wide mapping of global-to-local genetic effects on human facial shape. *Nat. Genet.* **50**, 414 (2018).

12. Horn, J. L. A rationale and test for the number of factors in factor analysis. *Psychometrika* (1965) doi:10.1007/BF02289447.
13. Hayton, J. C., Allen, D. G. & Scarpello, V. Factor Retention Decisions in Exploratory Factor Analysis: a Tutorial on Parallel Analysis. *Organ. Res. Methods* **7**, 191–205 (2004).
14. Claes, P. *et al.* Computerized craniofacial reconstruction: Conceptual framework and review. *Forensic Science International* (2010) doi:10.1016/j.forsciint.2010.03.008.
15. Claes, P. *et al.* Bayesian estimation of optimal craniofacial reconstructions. *Forensic Sci. Int.* (2010) doi:10.1016/j.forsciint.2010.03.009.
16. Claes, P. *et al.* The normal-equivalent: A patient-specific assessment of facial harmony. *Int. J. Oral Maxillofac. Surg.* (2013) doi:10.1016/j.ijom.2013.03.011.
17. Moreau, Y. Crack down on genomic surveillance. *Nature* **576**, 36–38 (2019).
